# Foundation models in omics research: a comprehensive survey

**DOI:** 10.1093/bib/bbag439

**Published:** 2026-08-19

**Authors:** Haozhe Liu, Wenhao Cai, Yizheng Sun, Haiping Liu, Zhiyong Zou, Qian Zhao, Sokratia Georgaka, Hongpeng Zhou, Jingyuan Sun

**Affiliations:** Department of Computer Science, The University of Manchester, Oxford Road, Manchester M13 9PL, United Kingdom; Department of Computer Science, The University of Manchester, Oxford Road, Manchester M13 9PL, United Kingdom; Department of Computer Science, The University of Manchester, Oxford Road, Manchester M13 9PL, United Kingdom; Department of Computer Science, The University of Manchester, Oxford Road, Manchester M13 9PL, United Kingdom; Division of Developmental Biology and Medicine, Faculty of Biology, Medicine and Health, The University of Manchester, Oxford Road, Manchester M13 9PL, United Kingdom; Division of Informatics, Imaging and Data Sciences, Faculty of Biology, Medicine and Health, The University of Manchester, Oxford Road, Manchester M13 9PL, United Kingdom; Division of Informatics, Imaging and Data Sciences, Faculty of Biology, Medicine and Health, The University of Manchester, Oxford Road, Manchester M13 9PL, United Kingdom; Department of Computer Science, The University of Manchester, Oxford Road, Manchester M13 9PL, United Kingdom; Department of Computer Science, The University of Manchester, Oxford Road, Manchester M13 9PL, United Kingdom

**Keywords:** foundation models, Transformer architectures, cross-modal learning, biological data representation, multi-omics analysis

## Abstract

The rapid expansion of high-throughput omics has created molecular datasets of unprecedented scale and complexity. These data are rich in biological information yet inherently sparse and high-dimensional, often limiting the effectiveness of conventional machine learning techniques. Foundation models (FMs), built on large-scale self-supervised pretraining, offer a robust alternative by learning generalizable representations directly from raw biological data. This review systematically analyzes the emerging landscape of FMs in omics research, spanning sequence modeling, cell state characterization, and multimodal integration. We organize the current literature into three distinct paradigms—sequence-centric, cell-centric, and multi-omics—to clarify a field currently fragmented by diverse tokenization strategies and architectural choices. Beyond methodology, we evaluate the practical utility of these models in tasks ranging from biomarker discovery to perturbation response prediction. We also identify critical barriers to adoption, including high computational costs, interpretability challenges, and the lack of standardized benchmarks. To support reproducible research, we provide a curated catalog of essential datasets and evaluation frameworks. Finally, we propose a roadmap for the next generation of FMs, advocating for architectures that move beyond statistical correlation to incorporate causal reasoning, temporal dynamics, and autonomous experimental validation.

## Introduction

The rapid evolution of high-throughput sequencing technologies has fundamentally transformed omics research. These advancements have generated vast quantities of single-omics data—spanning genomics (e.g. gnomAD [1]), transcriptomics (e.g. Human Cell Atlas (HCA) [2]), and proteomics (e.g. Human Protein Atlas (HPA) [3], UniProt [4])—that serve as the bedrock for understanding biological processes and disease mechanisms. Concurrently, the field of multi-omics seeks to capture system-level insights by integrating these diverse molecular modalities. However, the analysis of such data faces significant bottlenecks. Traditional statistical and machine learning approaches, which often rely on handcrafted features, struggle to generalize across different data scales and modalities. Furthermore, the intrinsic characteristics of omics data—including high dimensionality, sparsity, label scarcity, and cross-modality discrepancies—exacerbate data quality issues and amplify the risk of overfitting [5, 6]. These inherent limitations underscore the urgent need for flexible, self-supervised frameworks capable of modeling the complex, nonlinear structures governing biological systems.

Before the emergence of foundation models (FMs), conventional and task-specific machine learning and deep learning methods played a foundational role in omics research. In early genomics applications, traditional machine learning methods relied on representing genome-wide expression profiles as high-dimensional feature matrices and applying algorithms such as unsupervised clustering (hierarchical clustering and self-organizing maps) and supervised classification (weighted-voting classifiers) to identify molecular subtypes and predict disease classes [7, 8]. A second phase emerged with the rise of deep learning, in which multilayer neural networks enabled hierarchical representation learning from raw or minimally processed biological data. One major development in this phase was the use of convolutional neural networks (CNNs), which shifted genomic analysis from manually designed features toward end-to-end feature learning. Representative models such as DeepBind, DeepSEA, and DeepChrome learned local sequence motifs or combinatorial epigenetic patterns directly from genomic and epigenomic data, supporting protein-binding prediction, functional annotation of noncoding variants, and gene-expression prediction [9–11]. Another important extension was the application of graph neural networks to relational biological structures, where information could be propagated across cell–cell, sample–sample, or molecular interaction graphs. For example, scGNN modeled cell–cell relationships for single-cell RNA-sequencing (scRNA-seq) analysis, whereas MOGONET used graph convolutional networks to integrate multiple omics modalities for biomedical classification and biomarker identification [12, 13]. Attention mechanisms were subsequently incorporated into convolutional, recurrent, and graph-based architectures. AttentiveChrome employed hierarchical recurrent attention to identify informative genomic positions and histone modifications for gene-expression prediction, while DeepTACT combined convolutional feature extraction, bidirectional recurrent layers, and attention to predict chromatin contacts between regulatory elements [14, 15]. Within graph-based learning, graph attention networks enabled models to assign different importance to neighboring nodes or molecular relationships; representative applications include CellVGAE for single-cell representation learning and clustering and MODIG for integrating multi-omics profiles with multidimensional gene networks to identify cancer driver genes [16, 17]. Collectively, these developments progressively reduced reliance on handcrafted features and enabled increasingly complex sequence, epigenetic, and relational structures to be modeled. Nevertheless, most of these methods remained purpose-built for predefined tasks, modalities, and input structures, often requiring task-specific supervision, modality-dependent preprocessing, explicit graph construction, or substantial retraining when transferred to new datasets and biological contexts.

More importantly, these challenges are not independent, but tightly coupled in omics data. High dimensionality and sparsity make it difficult to learn stable task-specific predictors, while label scarcity prevents supervised models from fully exploiting increasingly large biological repositories. In multi-omics settings, discrepancies in resolution, feature space, and noise characteristics further complicate direct integration across modalities. The significance of FMs lies precisely in their ability to help mitigate these challenges through unified pretraining mechanisms. Self-supervised objectives such as masked modeling allow models to learn from incomplete and unlabeled data by reconstructing missing biological signals from context, thereby improving robustness to sparsity and reducing reliance on annotations. Large-scale representation learning compresses high-dimensional molecular inputs into transferable latent spaces that can be adapted across tasks. In turn, attention-based fusion and contrastive alignment provide a principled way to connect heterogeneous omics modalities, enabling models to capture cross-modal dependencies that are difficult to engineer manually. From this perspective, the advantage of FMs in omics is not merely that they are larger models, but that their core learning mechanisms are naturally matched to the intrinsic statistical structure of biological data.

Recent advances in FMs provide a promising framework to these challenges. Rather than functioning as isolated algorithmic innovations, FMs represent a paradigm shift toward end-to-end learning based on large-scale, self-supervised pretraining. By leveraging biological datasets, FMs extract generalizable representations of molecular systems that can be effectively transferred to diverse downstream tasks [18]. Pioneering models such as BERT [19], GPT [20], and CLIP [21] have demonstrated that architectures with billions of parameters can learn highly transferable features from heterogeneous data, a capability evidenced by their transformative impact on natural language processing (NLP) and computer vision [22–24].

The applicability of FMs to biology is rooted not only in their architectural scale, but also in the close match between their learning mechanisms and the intrinsic statistical properties of omics data. Self-attention enables context-aware modeling of high-dimensional molecular features, masked self-supervision allows training on sparse and partially observed data, and large-scale pretraining reduces dependence on costly annotations. These properties make FMs particularly well suited for single-omics data, where abundant genomic, transcriptomic, and other modality-specific resources can be used to learn robust biological representations without extensive manual feature engineering. Building on this foundation, FMs can be further adapted to multimodal settings, in which cross-attention and contrastive objectives provide flexible mechanisms for aligning heterogeneous omics modalities despite differences in feature space, resolution, and noise profile. This design facilitates the integration of diverse biological modalities more effectively than traditional feature-based methods [25]. Collectively, these capabilities—robust representation learning, scalable pretraining, and cross-modal integration—position FMs as a powerful foundation for alleviating several long-standing analytical hurdles in omics research [18].

Building on this potential, recent studies have successfully extended FMs to both single-omics and multi-omics domains. These advances are not merely application-specific; rather, they are enabled by concrete choices in tokenization, self-supervised objectives, and architecture design that allow FMs to learn transferable biological representations across omics modalities. In the single-omics sphere, FMs have been adapted for genomics, proteomics, and spatial transcriptomics [5, 26]. For instance, in genomics the Nucleotide Transformer employs k-mer tokenization and masked pretraining to capture contextual dependencies in DNA sequences, enabling the model to learn transferable representations that support tasks such as regulatory element identification and mutation effect prediction [27]. In proteomics, protein language models such as ESM [28] and ProtT5 [29] are pretrained on massive amino acid sequence corpora, allowing them to encode residue-level context and higher-order biochemical regularities that can be transferred to protein function and pathogenicity prediction. In transcriptomics, models that treat genes as tokens, including scGPT [30] and Geneformer [31], leverage large-scale pretraining on single-cell expression profiles to learn context-aware cellular representations, thereby improving downstream tasks such as cell-type annotation and perturbation response modeling. Similarly, Nicheformer [32] uses joint pretraining on dissociated and spatial RNA profiles to align cellular states with spatial context, illustrating how FMs can integrate complementary views of biological organization to reconstruct tissue architecture. Although these examples demonstrate the effectiveness of FMs within individual omics layers or closely related data types, biological states and disease phenotypes typically arise from coordinated interactions among multiple molecular layers. This motivates a natural extension from modality-specific representation learning toward multi-omics FMs. By leveraging complementary signals and structured dependencies across molecular layers, multi-omics FMs can construct more comprehensive representations of biological states and enable the inference of unobserved or incompletely measured modalities.

To realize these benefits, multi-omics FMs generally adopt two complementary modeling strategies: shared-representation integration and cross-modal prediction or inference. Shared-representation integration projects heterogeneous modalities into a common latent space, enabling modality-specific observations to be compared and jointly modeled. This computational choice is biologically motivated by the fact that genomic, epigenomic, transcriptomic, proteomic, and histopathological measurements provide distinct but complementary views of the same underlying cellular or disease state. Aligning these modalities therefore enables the model to capture coordinated biological programs that may not be apparent from any single data type alone. For example, CLOVER [33] uses contrastive learning to align pathology whole-slide images with CNV, methylation, and transcriptomic profiles, thereby learning cross-modal representations that support cancer subtype classification. Cross-modal prediction or inference, by contrast, learns relationships between modalities so that an unobserved or incompletely measured molecular layer can be inferred from one or more available modalities. This strategy is biologically justified by the structured dependencies among molecular layers, including the relationships among genomic variation, epigenetic regulation, chromatin organization, transcription, and protein activity. Because changes in one layer can constrain or provide information about another, models can exploit these dependencies to reconstruct missing or experimentally difficult-to-obtain measurements. IsoFormer [34], for instance, fuses DNA, RNA, and protein modalities through cross-modal attention to predict transcript abundance, while HiCFoundation [35] leverages representations learned from Hi-C data to infer chromatin accessibility and protein-binding tracks. Conceptually, shared-representation integration identifies biological information that is common or complementary across modalities, whereas cross-modal prediction captures how information in one molecular layer is informative of another. Together, these strategies demonstrate the capacity of FMs to model the coordinated, multi-level organization of biological systems.

Despite this rapid progress, the existing literature remains fragmented. Most prior reviews focus either on general machine learning approaches for multi-omics [36, 37] or are not specifically tailored to FMs [38–40], rarely synthesizing FM perspectives across the broader omics landscape. To address this gap, we provide a focused review of FMs in omics research. We begin by outlining the relevant omics modalities and datasets. We then systematically examine methodological paradigms for developing FM-based models along three complementary axes: sequence-centric, cell-centric, and multi-omics models. Next, we review prominent applications and state-of-the-art models, followed by a conceptual guide to FM training. Finally, we analyze existing methodological and practical challenges and propose a roadmap for future research in this emerging field.

## Overview of omics modalities and multi-omics data resources

The development of FMs for biological data requires a clear understanding of the molecular layers that constitute the omics landscape and the datasets that underpin them. Omics technologies quantify distinct but interconnected aspects of cellular systems—including genomic sequence, epigenetic regulation, RNA expression, protein abundance, and downstream metabolites—forming a hierarchical representation of biological information that follows the central dogma [41, 42]. Each modality captures a different facet of cellular state, and jointly they provide the multidimensional signals essential for constructing biologically coherent FM representations.

Because these molecular layers underpin nearly all computational analyses in modern biology, understanding their corresponding data resources is essential for developing and evaluating FMs.

This section provides a structured overview of major omics resources, grouped according to the biological layer or analytical role they represent—genomics and epigenomics, transcriptomics, proteomics and structural biology, genetics and phenomics, functional genomics, and multi-omics cohorts—reflecting both the flow of information in molecular biology and the data hierarchies most relevant for FM pretraining, genotype-to-phenotype modeling, and cross-modal alignment. Representative examples of each category are described in the subsections below, with a more comprehensive list summarized in Table 1.

**Table 1:** Summary of omics datasets and resources.

**Omics type**	**Resource**	**Data type**	**Technical characteristics (assays/formats/scale)**	**Description**	**URL**
Genomics & epigenomics	GENCODE [43]	Gene annotation	Hybrid Ensembl+HAVANA manual curation; GTF/GFF3/FASTA; 78 691 human genes	Reference gene annotations for human and mouse genomes.	Link
	Ensembl [49]	Integrated genomic platform	Aggregated pipelines across >50 000 species; GTF/GFF3/FASTA/VCF; >4800 eukaryotic genomes	Automated access to genome sequence, variation, regulation and comparative genomics.	Link
	UCSC Genome Browser [50]	Visualization & database	Hosts ENCODE/GENCODE and external tracks; BED/WIG/GTF/bigWig; *∼ *65 000 annotation tracks	Interactive web interface for exploring genome assemblies and aligned functional tracks.	Link
	ENCODE [51]	Regulatory elements	ChIP-seq, DNase-seq, RNA-seq, CAGE; FASTQ/BAM/bigWig/bigBed/hic; *∼ *106k datasets	Open-access maps of chromatin accessibility, TF binding, DNA methylation and transcription.	Link
	Roadmap Epigenomics [46]	Epigenomic landscapes	Primary-tissue ChIP-seq & bisulfite-seq; BAM/BED/bigWig/tagAlign; 127 consolidated epigenomes	Genome-wide maps of histone marks, accessibility, methylation and expression with unified processing.	Link
	Ensembl Regulatory Build [45]	Regulatory annotation	Integration of ENCODE/Roadmap functional data; GFF/BED/bigBed/JSON; 643 528 GRCh38 features	*In silico* predictions of promoters and enhancers from public regulatory data.	Link
	FANTOM 5/6 [52, 53]	Transcriptional regulation	CAGE promoter/enhancer profiling; CTSS/BED/TSV; >3000 human and mouse samples	Expression atlases of promoters, enhancers and lncRNAs across diverse cell types.	Link
	EnhancerAtlas 2.0 [54]	Enhancer–gene interactions	Consensus across multi-omics studies; BED; 13 494 603 enhancers	Repository of tissue-specific enhancers and predicted enhancer–target pairs in nine species.	Link
	EPD [55]	Pol II promoters (TSS)	CAGE/TSS-seq–based screening; FASTA/BED; 15 species	Curated set of experimentally validated eukaryotic promoters with precise transcription start sites (TSS) locations.	Link
	RefSeq [56]	Nonredundant reference sequences	Curation from GenBank submissions; FASTA/GFF3/GBFF; 170 401 organisms	Stable, nonredundant and well-annotated reference genomes, transcripts and proteins for many species.	Link
	GEO [57]	Functional genomics data	Submitter-driven archive of array/seq studies; SOFT/Excel/MINiML; >8.1M samples	Public functional genomics data repository for MIAME-compliant microarray and sequencing experiments.	Link
	OpenGenome2 [58]	Full-domain genomic corpus	Multi-kingdom aggregation from public sources; JSONL/FASTA; 8.8T bp	Large-scale genomic corpus from different species and public data for training Evo2 and related FMs.	Link
Transcriptomics	RNAcentral [47]	Noncoding RNA sequences	Aggregated expert databases; FASTA/BED/GFF3/TSV; >22M secondary structures	Unified identifiers and sequences for ncRNAs collected from >50 specialist resources.	Link
	Rfam [48]	RNA families & structure models	Seed alignments & covariance models; Stockholm/FASTA/CM/BED; 4178 families	Library of RNA families with consensus structures and HMM/CM profiles.	Link
	HCA [2]	Cellular Reference Maps	Community-based Multi-omics Collection; Loom, H5AD; 70.3M cells	A comprehensive reference maps of all human cells to support insights into health, disease mechanisms, and therapeutic development.	Link
	Single Cell Portal (SCP) [59]	scRNA-seq studies	Researcher-submitted datasets; MTX/TSV; 926 studies, 70M+ cells	Broad Institute platform for sharing and interactive visualization of single-cell data.	Link
	Genecorpus-30M [31]	Pretraining gene corpus	Large-scale gene-sequence aggregation; Apache Arrow (HF datasets); 30M gene sequences	Tokenized gene corpus tailored for training models such as Geneformer.	Link
	SpatialCorpus-110M [32]	Spatial transcriptomics	Multi-technology aggregation; AnnData (.h5ad); 110M+ cells from 17 organs	10M+ single-cell profiles across 17 organs and 18 cell lines from human and mouse, with additional data from other anatomical systems.	Link
	10x Genomics datasets [60]	Single-cell & spatial multi-omics	Droplet microfluidics & spatial barcoding; FASTQ/feature–barcode matrices (.mtx/.h5); 702 public datasets	Manufacturer-hosted repository of Chromium, Visium and Xenium single-cell/spatial runs.	Link
	Basenji functional tracks [61]	Regulatory activity prediction	CNN-ready functional tracks; HDF5/TensorFlow; *∼ *6000 experiments	Sequence–to–signal datasets for training Basenji models on genome-wide regulatory profiles.	Link
Proteomics & structure	UniProt [4, 62]	Protein sequence & function	Swiss-Prot curation + TrEMBL translation; FASTA/tab/GFF/XML; 573 661 reviewed entries	Global core resource of functionally annotated protein sequences.	Link
	Gene Ontology (GO) [63]	Functional vocabulary & annotation	Manual curation + automated inference; OBO/OWL/GAF; 9.3M annotations	World’s largest repository of standardized ontology and annotations describing molecular function, process and location.	Link
	Pfam [64]	Protein domains & families	HMMs from seed alignments; Stockholm/HMM; 21 979 families	Domain/family models for annotating proteins using profile HMMs.	Link
	RCSB PDB / PDBj / PDBe [65–67]	3D macromolecular structures	X-ray, NMR, cryo-EM; PDB/PDBx/mmCIF; 245 778 entries	Central archive of experimentally determined and computed macromolecular structures.	Link
	BMRB [68]	NMR spectral data	Solution-state NMR experiments; NMR-STAR; >11 900 entries	Quantitative NMR measurements for biomacromolecules and metabolites.	Link
	EMDB [69]	3D density maps	Cryo-EM reconstruction; CCP4/MRC; 51 712 maps	Open repository of cryo-EM volumes and tomograms for macro- and subcellular structures.	Link
	Human Protein Atlas (HPA) [3]	Spatial protein expression	IHC/IF-based profiling; images/TSV/XML; 27 883 antibodies, 17 407 proteins	Atlas of protein localization across tissues, cell types and subcellular compartments.	Link
Genetics & phenomics	dbSNP [70]	Genomic variation	Community submissions + automated aggregation; VCF/JSON; 4.4B submitted records	Catalog of SNVs, microsatellites and indels with population and functional context.	Link
	gnomAD [1]	Population allele frequencies	Harmonized exome/genome calling; VCF; 807 162 individuals	Integrated exome and genome sequencing resource comprising 730 947 exomes and 76 215 whole genomes (v4, GRCh38) from unrelated individuals spanning diverse ancestries	Link
	ClinVar [71]	Variant clinical significance	Clinical lab & expert submissions; XML/VCF; 92 568 genes represented	Clinically interpreted variants and phenotypes with evidence graded by a ’Star System’.	Link
	GWAS Catalog [72]	GWAS summary statistics	Literature curation & community deposition; GWAS-SSF; 135 378 studies	Curated SNP–trait associations and full-summary statistics from human GWAS.	Link
	UK Biobank [73]	Genotype & phenotype cohort	Prospective cohort with linked EHR/imaging; mixed formats (CSV/DICOM etc.); 500 000 participants	Deeply phenotyped cohort with genetic, imaging and health record data.	Link
	FinnGen [74]	Genotype & registry data	National biobank network; VCF/REGENIE/GWAS-SSF; 500 348 donors	Finnish genome–health resource for disease gene discovery and risk prediction.	Link
Functional genomics	DepMap (Achilles/DRIVE) [75–77]	Gene dependency / essentiality	Genome-wide CRISPR & RNAi screens; CSV/TSV; 1275 cancer cell lines	Catalog of cancer gene dependencies with matched molecular profiles.	Link
	DepMap-PRISM [75]	Drug sensitivity (AUC/IC50)	DNA-barcoded pooled viability screens; CSV/TSV; 4518 drugs *× * 578 cell lines	Multiplexed chemical-perturbation screen for repurposing and mechanism studies.	Link
	GDSC [78]	Drug sensitivity (IC50/AUC)	Arrayed high-throughput assays; CSV; *∼ *1000 cancer cell lines	Drug–response measurements linked to mutational, CNV and expression profiles.	Link
	CCLE [78]	Cell-line multi-omics	WES/WGS/RNA-seq/RRBS; CSV/TSV; 1019 lines	Comprehensive genomic and epigenomic characterization of cancer cell lines.	Link
	CTRP [79]	Drug sensitivity (AUC)	Arrayed CTG viability assays; CSV/XML; 481 compounds *× * 860 CCLs	Library of tool compounds for linking small molecules to pathway dependencies.	Link
	LINCS L1000 [80]	Transcriptional signatures	L1000 bead-based assay; GCT/GCTX/CSV; 978 landmark genes, 216 105 inferred	High-throughput perturbation profiles inferred from a reduced gene set.	Link
	Connectivity Map (CMAP) [81]	Perturbation signatures	Pattern-matching over L1000 data; GCT/GCTX; >1.5M profiles	Cloud platform for querying gene-expression responses to genetic and chemical perturbations.	Link
Multi-omics	OpenProblems [82]	Benchmarking tasks & datasets	Community challenges; AnnData (.h5ad); 12 single-cell tasks	Defines tasks, curated train/test data and unified metrics for omics method benchmarking.	Link
	TCGA [83]	Multi-omics & clinical outcomes	Matched tumor–normal sequencing; mixed formats; >20 000 samples, 33 cancer types	Pan-cancer resource of DNA, RNA, epigenetics and clinical data.	Link
	CPTAC [84]	Proteogenomics (protein/phospho)	LC–MS/MS + genomics; TSV/BAM/VCF/MAF/HDF5; 1500+ patients	Integrative proteomic and genomic characterization of multiple tumor types.	Link
	ICGC 25k [85]	Pan-cancer genomes	International whole-genome projects; TSV/BAM/VCF; >20 000 tumors (26+ cancer types)	Global effort sequencing *∼ *25k cancer genomes; PCAWG is a 2800-sample high-quality subset.	Link
	METABRIC (EGA) [86]	Breast-cancer multi-omics	SNP and expression microarrays; TSV/CEL; 2509 primary tumors	Gold-standard breast-cancer cohort with long-term clinical follow-up for survival analyses.	Link
	GTEx [87]	Multi-tissue eQTLs	Post-mortem multi-tissue WGS + RNA-seq; GCT/VCF/TSV; *∼ *17 382 RNA-seq samples	Links genetic variants to gene-expression variation across 54 human tissues.	Link
	scIB [88]	Benchmarking metrics	Systematic scRNA-seq integration tests; Python package; 85 batches	Framework for comparing single-cell integration methods using harmonized metrics.	Link

*Note*: Resources are listed with their primary data modalities. Summarizes representative, widely used, and publicly accessible omics datasets and resources relevant to foundation model development. Given the breadth and rapid expansion of omics data, this table is intended as an illustrative rather than exhaustive compilation.

### Genomics and epigenomics resources

Genomic and epigenomic resources form the foundation of sequence-based modeling and regulatory inference. Core reference datasets such as *GENCODE* [43] provide curated gene models, transcript structures, and biotype annotations that establish the canonical coordinate system for downstream analysis. Regulatory mapping efforts—including *ENCODE (ENCyclopedia Of DNA Elements)* [44], *Ensembl Regulatory Build* [45], and *Roadmap Epigenomics Consortium* [46]—offer uniformly processed profiles of chromatin accessibility, histone modifications, and transcription factor binding across diverse tissues and cell types. These datasets enable the annotation of promoters, enhancers, and other regulatory elements, supporting both variant interpretation and sequence-to-function modeling. Their consistent formats (e.g. FASTA, GTF/GFF, BED, bigWig) and broad coverage make them indispensable for pretraining sequence-based FMs and for studying cross-cell-type regulatory dynamics.

### Transcriptomics resources

Transcriptomics captures the dynamic state of gene activity and is central to modeling cellular function. Bulk RNA-seq resources, noncoding RNA repositories such as *noncoding RNA sequence database (RNAcentral)* [47] and *RNA Familie (Rfam)* [48], and increasingly comprehensive single-cell RNA-seq datasets provide rich corpora for learning expression-level representations. These datasets differ substantially in noise profile, sparsity, and granularity, offering complementary training signals for models that treat genes as tokens or that learn embeddings of cell states. Long-read sequencing and spatial transcriptomics further expand transcript diversity and contextual resolution, enabling models to bridge sequence variation, isoform structure, and spatial cellular organization.

### Proteomics and structural biology resources

Protein-centered resources underpin models of molecular function and structure. Curated sequence repositories such as *Universal Protein Resource (UniProt)* [4] and domain-level catalogs like *The protein families database (Pfam)* [64] provide high-quality annotations of motifs, domains, and posttranslational modifications. Structural datasets—including the *Protein Data Bank (PDB)* [89], *Biological Magnetic Resonance Data Bank (BMRB)* [68], and *Electron Microscopy Data Bank (EMDB)* [69]—offer experimentally determined 3D conformations essential for learning geometric and physicochemical representations. These resources support a wide range of FM applications, from protein folding and design to variant effect prediction, and benefit from mature standardized formats (e.g. FASTA, mmCIF, MSA files).

### Genetics and phenomics resources

Genetics and phenomics resources connect molecular variation to organism-level traits, disease phenotypes, and population diversity, thereby extending omics modeling beyond molecular measurement toward clinically and biologically meaningful outcomes. Variant-centric databases such as *dbSNP* [70], *gnomAD* [1], and *ClinVar* [71] provide large-scale catalogs of genetic variation, allele frequency, and clinical interpretation, supporting tasks such as rare-variant prioritization, pathogenicity prediction, and population-aware representation learning. In parallel, association resources such as the *GWAS Catalog* [72] and large biobank-scale cohorts including *UK Biobank* [73] and *FinnGen* [74] link genotypes with complex traits, imaging, electronic health records, and longitudinal disease outcomes. These datasets are particularly valuable for training FMs aimed at genotype-to-phenotype prediction, disease risk stratification, and multimodal clinical representation learning, while also providing an important bridge between molecular omics and real-world biomedical phenotypes.

### Functional genomics and perturbation screens

Functional genomics datasets capture causal relationships between genetic perturbations and cellular phenotypes. Large-scale CRISPR and RNAi screening efforts—such as *Dependency Map (DepMap)* [76], *Cancer Therapeutics Response Portal (CTRP)* [79], and *Genomics of Drug Sensitivity in Cancer (GDSC)* [78]—link gene essentiality, drug response, and molecular features across panels of cell lines. High-throughput perturbation assays combined with single-cell readouts (e.g. Perturb-seq) provide fine-grained measurements of gene regulatory effects and pathway-level interactions. These datasets are particularly valuable for training FMs to model causal and mechanistic relationships, moving beyond correlation-based inference.

### Multi-omics cohorts

Comprehensive cohorts that jointly profile multiple omics layers from matched samples provide a natural substrate for cross-modal learning. Initiatives such as *The Cancer Genome Atlas (TCGA)* [83], *Clinical Proteomic Tumor Analysis Consortium (CPTAC)* [84], *International Cancer Genome Consortium (ICGC)* [85], and *UK Biobank* [73] integrate genomic variation, transcriptomic expression, proteomic abundance, epigenetic marks, medical images, and clinical phenotypes. These resources enable models to learn relationships across modalities, support cross-omics imputation and alignment tasks, and provide benchmarks for multimodal representation learning. Their harmonized structure and standardized preprocessing pipelines make them central to evaluating FM generalization in real-world biomedical settings.

## Methodological paradigms in omics foundation models

**Figure 1:**
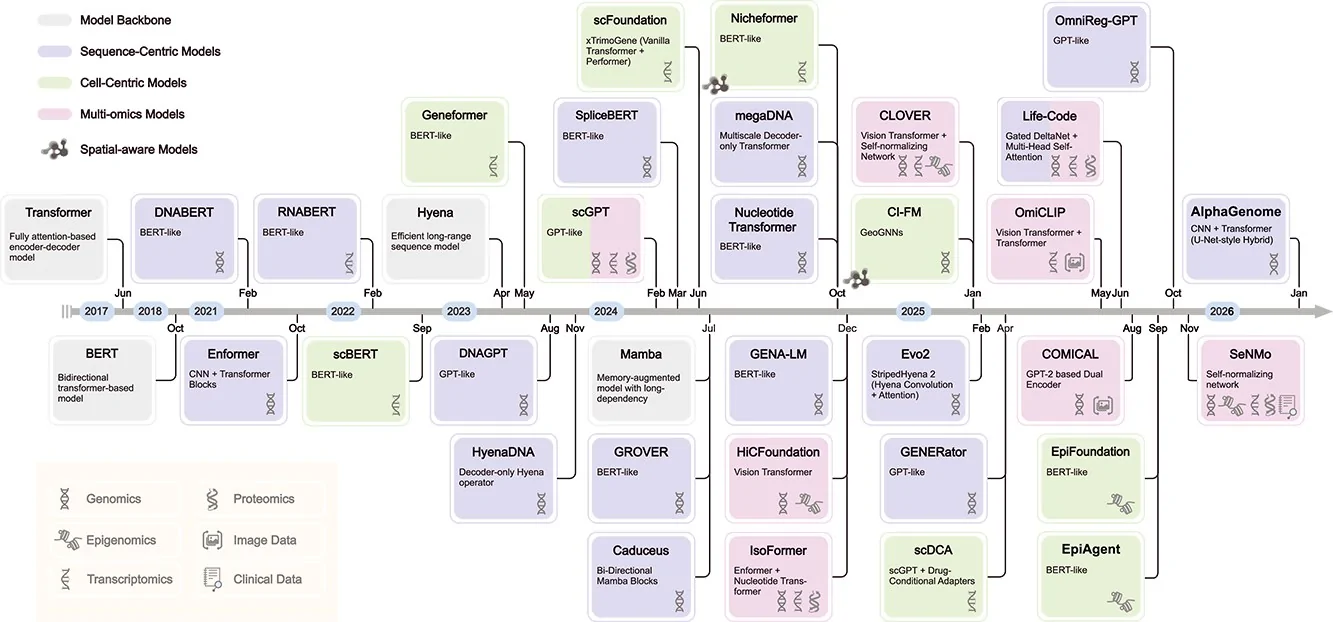
Chronological landscape of omics FMs, grouped by methodological category (color-coded) and annotated with the biological data modalities they leverage, reflecting the field's evolution toward increasingly diverse and integrated modalities as multi-omics technologies expand.

FMs for biological data vary widely in how they represent molecular entities, structure biological knowledge, and integrate heterogeneous signals. As omics resources span sequences, cellular measurements, and multilayered molecular readouts, different modeling paradigms have emerged to capture the distinct structure of these data (Fig. 1). Importantly, these paradigms are not merely computational design choices, but reflect different biological assumptions regarding the organization of molecular systems, ranging from sequence-level encoding of regulatory information to cell-level state representations and cross-modal interactions.

**Figure 2:**
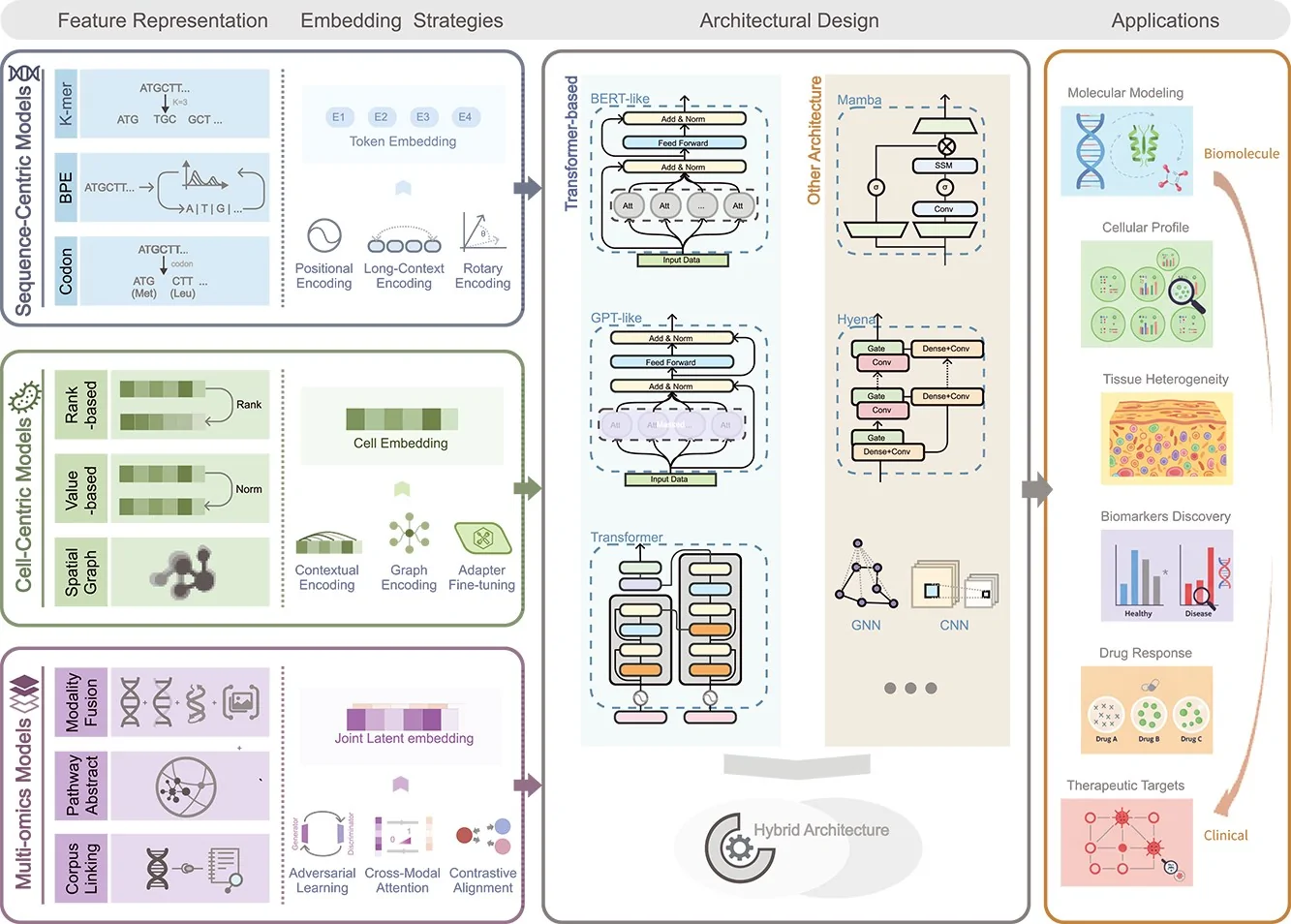
Schematic of omics FM architectures and their downstream applications. Omics FMs can be broadly categorized as sequence-centric, cell-centric, or multi-omics, yet they generally follow a common pipeline comprising several interacting components: representing biological inputs, applying embedding strategies, and selecting network architectures. Downstream analyses span multiple biological scales, from molecular and cellular levels to tissue-level and clinical tasks.

Despite substantial architectural diversity, current omics FMs can be broadly understood through three conceptual paradigms corresponding to different levels of biological organization: sequence-centric, cell-centric, and multi-omics FMs (Fig. 2). Sequence-centric models operate directly on molecular sequences, such as DNA, RNA, or proteins, treating them as the fundamental substrates from which biological structure and function emerge. Cell-centric models instead center on cells, representing their gene expression states as well as their spatial, developmental, or functional relationships. Multi-omics models further extend this perspective by integrating multiple molecular layers into a unified representational space, with the goal of capturing cross-modal dependencies that underlie complex biological phenotypes. Importantly, these paradigms can also be interpreted from a shared problem-oriented perspective: although they differ in biological input and representation level, they all aim to help mitigate these challenges the core challenges of omics data—including high dimensionality, sparsity, limited labels, and cross-modal heterogeneity—through distinct representation strategies and self-supervised objectives. Accordingly, this taxonomy serves not only as a classification by biological input type, but also as a framework for understanding how FM mechanisms are adapted to the intrinsic challenges of different omics settings.

These paradigms share the overarching goal of learning generalizable representations from large biological corpora, yet they differ in their inductive biases, feature representation strategies, architectural assumptions, and typical pretraining signals. Crucially, these differences correspond to distinct biological hypotheses regarding how information is structured and propagated in living systems, such as whether regulatory signals are locally encoded in sequences, globally organized in cellular states, or distributed across molecular modalities. Understanding these differences is essential for interpreting how FMs encode biology and for situating the rapidly growing literature within a coherent methodological landscape.

In the following subsections, we outline the defining representational choices and modeling strategies characteristic of each paradigm, and discuss representative examples that illustrate how these principles are implemented in practice.

### Sequence-centric foundation models

Sequence-centric FMs represent the earliest and most direct extension of natural language modeling to biology field [90]. These models conceptualize DNA, RNA, or protein sequences as linguistic entities composed of discrete symbols, enabling the transfer of tokenization and embedding strategies from NLP [91, 92]. This analogy is grounded in the biological observation that molecular sequences consist of discrete functional elements (e.g. motifs and regulatory regions) that carry intrinsic biological meaning, while their interactions and dependencies form complex regulatory relationships, analogous to semantic and syntactic structures in natural language.

Representation strategies typically begin by tokenizing sequences, evolving from simple k-mer tokenization, as used in DNABERT [93] and Nucleotide Transformer [27], to nucleotide-level tokenization adopted by models such as HyenaDNA [91, 94] and Evo2 [58], which preserve single-base resolution and maintain full sequence fidelity. More sophisticated data-driven schemes, including byte-pair encoding (BPE) in GENA-LM [95] and codon-level representations in Life-Code [41], further incorporate biological priors by aligning tokens with functional units such as codons and amino acids.

These tokenization strategies reflect different assumptions about the fundamental units of biological information: k-mer and BPE methods emphasize statistical regularities, nucleotide-level tokenization preserves maximal sequence fidelity, while codon-level representations introduce explicit biological semantics. However, these design choices introduce an inherent trade-off between biological resolution and computational efficiency. Fine-grained tokenization such as nucleotide-level results in significantly longer input sequences, increasing memory and computational costs during training and limiting effective context length, whereas coarser tokenization such as k-mer reduces sequence length and improves scalability at the expense of potentially losing precise positional or functional information.

Once tokenized, sequences are projected into dense latent spaces through learned embeddings combined with positional or rotary encodings that preserve contextual order along the molecular sequence [96]. Through pretraining on large-scale sequence corpora, these latent representations can capture complex molecular dependencies and are often interpreted as encoding regulatory relationships.

Architecturally, Transformers dominate sequence-centric FMs due to their ability to model complex dependencies between sequence elements through pair-wise attention blocks. From a biological perspective, these mechanisms can be viewed as approximating regulatory interactions, where highly weighted connections may correspond to regulatory hubs or co-functional sequence elements. In encoder-only architectures (e.g. DNABERT [93], Nucleotide Transformer [29]), each unmasked token attends bidirectionally to both upstream and downstream sequence elements. During masked pretraining, selected tokens are removed or corrupted, and the model reconstructs them from the remaining context. This process produces context-dependent token embeddings that capture local motifs, residue environments, domain organization, and longer-range sequence dependencies. These token-level representations can support regulatory-element annotation, residue-function prediction, and variant-effect estimation, while pooling across tokens yields sequence-level embeddings for classification and retrieval. Importantly, attention weights represent statistical dependencies learned by the model and should not be interpreted as direct evidence of regulatory or physical interactions without additional validation. Decoder-only architectures (e.g. OmniReg-GPT [97]) instead apply a causal attention mask, such that each token is predicted from preceding sequence tokens and any supplied conditioning variables. Next-token pretraining therefore learns an explicit conditional probability distribution over possible nucleotide, codon, or amino-acid continuations. This distribution can be used to score sequence likelihood, evaluate candidate mutations, or generate regulatory, coding, or protein sequences conditioned on properties such as species, promoter class, target expression, or protein function. Compared with encoder-only models, decoder-only architectures provide native generative capability, but use unidirectional context during prediction, require sequential generation, and may produce statistically plausible sequences that do not satisfy structural or functional biological constraints.

However, genomic regulatory signals can span millions of base pairs, making standard Transformers inefficient due to the quadratic computational cost of self-attention. As a result, early genome FMs are typically pretrained with limited context lengths, often ranging from 512 to 4096 tokens [91]. Although lower-resolution tokenization strategies such as k-mer are sometimes adopted to extend genomic coverage, these models still struggle to capture structured long-range dependencies arising from hierarchical regulatory processes such as enhancer–promoter interactions and chromatin folding due to architecture limitations.

To mitigate this, recent models explore alternative sequence modeling paradigms. State space models (e.g. Mamba) compress long-range context into continuous latent states, enabling efficient propagation of information across extremely long sequences while maintaining sensitivity to sequential order. In contrast, implicit convolution-based approaches (e.g. Hyena) use long convolutional filters to capture multi-scale dependencies, allowing the model to integrate both local motif patterns and distal regulatory signals in a more structured and translation-aware manner. These mechanisms provide inductive biases better aligned with biological processes, where regulatory effects are often distributed, hierarchical, and span large genomic distances. For example, Enformer[98] combines convolutional layers with self-attention, enabling joint modeling of local motifs and long-range regulatory interactions [98]. Models such as Caduceus [92] and Life-Code [41] take this idea further by incorporating biological inductive biases, such as enforcing reverse-complement symmetry and reflecting the central dogma. This shift moves the field from linguistic mimicry toward architectures that are grounded in biological reasoning.

Additionally, recent work has begun to extend sequence-centric models beyond isolated sequence representations by incorporating dynamic regulatory effects and higher-order biological context. For example, models such as AlphaGenome [99] capture long-range regulatory interactions and chromatin contact maps, enabling the modeling of dependencies across distant genomic loci and reflecting network-level organization of gene regulation. OmniReg-GPT [97] enables the modeling of regulatory sequences as spatially and temporally coordinated systems, supporting the prediction of context-dependent gene expression, chromatin accessibility, and 3D chromatin interactions. Together, these developments reflect a shift from generic sequence modeling toward architectures more closely aligned with biological principles.

Sequence-centric FMs have demonstrated strong performance in tasks such as regulatory element prediction, functional variant prioritization, RNA structure modeling, and protein representation learning. Sequence-centric FMs are particularly well suited to settings where biological meaning is distributed across extremely long, high-dimensional sequences and annotated labels are scarce. Their self-supervised pretraining objectives enable them to extract regulatory and structural regularities directly from raw sequence corpora, reducing reliance on handcrafted motif features while improving transferability across downstream genomic tasks, although they remain limited in representing context-dependent regulatory activity or interactions across higher biological layers.

### Cell-centric foundation models

Cell-centric FMs extend modeling from linear molecular sequences to the multidimensional, sparse, and heterogeneous profiles of individual cells. Here, the input unit is a cell, represented by gene expression, chromatin accessibility, spatial context, or multimodal measurements. Accordingly, the modeling objective shifts from learning sequence syntax to capturing the semantics of cellular states and transitions, including gene co-variation, tissue organization, and responses to perturbations [100].

Representation strategies generally fall into two categories: rank-based and value-based encodings. Rank-based approaches transform a cell’s expression profile into an ordered sequence of features, emphasizing relative importance as cell identity is typically characterized by differentially expressed or highly variable genes and coordinated regulatory programs. This design is particularly motivated by the intrinsic dropout events in scRNA-seq data, where absolute expression values are often noisy and unreliable, while relative gene expression patterns remain more stable across cells. For example, Geneformer [31] ranks genes by expression level to capture stable gene–gene relationships across datasets. Similarly, Nicheformer [32] organizes gene tokens based on relative expression ranks to mitigate the dominance of ubiquitously highly expressed genes, while enriching these tokens with contextual identifiers such as tissue, modality, or species to encode microenvironmental context. For epigenomic data such as scATAC-seq, models such as EpiAgent [101] also adopt rank-based strategies, ranking accessible cis-regulatory elements based on TF-IDF signals and capturing co-accessibility patterns that reflect chromatin-level regulatory networks. Overall, rank-based representations prioritize relative regulatory structure over absolute expression magnitude, improving robustness to technical variability such as library size and sequencing depth, but may discard quantitative expression differences.

In contrast, value-based approaches retain normalized expression values to preserve quantitative differences that may carry biological significance. This strategy aims to preserve the full magnitude information of cellular expression and is particularly relevant in scRNA-seq data, where the high sparsity leads to a concentration of informative signals in a subset of expressed genes, making the preservation of expression magnitude both computational feasible and informative for downstream modeling. Models such as scFoundation [102] adopt this strategy, directly encoding expression magnitudes to support tasks requiring precise quantitative modeling. Value-based representations preserve quantitative fidelity and enable fine-grained modeling of cellular states, but are more sensitive to technical variability such as batch effects.

For spatial omics data, cell-centric models further extend these representations by explicitly modeling cell–cell relationships. For example, CI-FM [60] represents cells as nodes in a graph connected by spatial or functional proximity, enabling direct modeling of cellular interactions within tissues. These representations map cellular features into continuous embedding spaces where attention mechanisms and neighborhood operators can reveal biological structure, such as gene–gene dependencies and cell–cell interactions.

Architecturally, cell-centric FMs build on the Transformer backbone but are adapted to reflect the biological structure of cellular systems [18]. Because cellular identity largely arises from coordinated gene expression, early cell-centric models are designed to capture intra-cellular regulatory relationships. In encoder-only architectures, gene identities or regulatory elements are combined with expression ranks, discretized abundance values, continuous measurements, or accessibility scores. Bidirectional attention allows each observed feature to be contextualized by the complete cellular profile. Under masked-gene or masked-value objectives, the model reconstructs hidden gene identities or molecular measurements from the remaining features. The resulting gene-level embeddings encode context-dependent co-expression or co-accessibility patterns, whereas a pooled representation or special cell token summarizes the global cellular state. These representations are particularly suitable for cell-type annotation, clustering, batch integration, phenotype prediction, and retrieval of transcriptionally similar cells. Decoder-only modeling is less straightforward for cell-centric omics because a cellular profile is naturally sparse and unordered rather than a 1D sequence. Autoregressive models must therefore introduce an ordering, such as expression-ranked genes or a fixed gene vocabulary, or iteratively predict unknown expression values conditioned on observed genes. When baseline cellular states are combined with perturbation, dose, time, tissue, or developmental-condition tokens, decoder-only models can generate condition-specific expression profiles or candidate post-perturbation states [103]. Their output is therefore a conditional distribution over possible cellular profiles rather than only a fixed embedding. For spatial omics, within-cell feature modeling is further extended with cross-cell context. Spatially aware models incorporate spatially resolved training data, neighborhood structure, coordinates, or graph-based interactions to represent tissue organization and the effects of local microenvironments on cellular phenotypes [32, 60].

Together, these designs move beyond traditional static dimensionality reduction toward context-aware representations of cell identity, interactions, and state transitions. As a result, cell-centric FMs achieve strong generalization across tasks such as cell-type annotation, perturbation modeling, batch integration, and spatial analysis. However, challenges remain, including cross-dataset variability, incomplete atlas coverage, the lack of scalable modeling and whole-slice pretraining strategies for spatial omics data, and limited ability to link cellular representations to upstream regulatory mechanisms without multimodal integration.

### Multi-omics foundation models

To advance toward a holistic understanding of biological systems and disease, integrative analysis of multi-omics data has become increasingly important, as different modalities capture complementary aspects of cellular state. Numerous tools have been developed for multi-omics study. Based on disease-related bulk multi-omics datasets, models such as DeePathNet [104], MOGONET [105], PCLSurv [106], CLCLSA [33], and CancerSD [107] achieve more refined cancer subtype classification and improved drug-response prediction through cross-omics integration. Among these, DeePathNet projects multi-omics features onto cancer-specific biological pathways to enable multimodal joint analysis [104], while MOGONET employs graph-based modality representations to capture structured cross-omic relationships [105]. In finer single-cell and spatial multi-omics field, GLUE [108] employs adversarial generative learning to align heterogeneous single-cell omics; SpatialGlue [109] introduces a dual-attention graph model to jointly analyze spatial multi-omics data generated from the same tissue section; and MultiGATE [110] as well as SpatialFuser [111] further extend these capabilities to achieve fine-resolution cross-slice integrative deciphering.

However, these models remain limited in general-purpose scalability, but provide valuable methodological insights that have informed the development of more recent integrative FMs, which aim to unify multiple omics modalities within coherent architectures that can reason across diverse biological scales. This paradigm recognizes that complex biological phenotypes arise from coordinated interactions across molecular hierarchies, and therefore seeks to jointly design representation and embedding strategies to encode cross-modal dependencies within a shared modeling framework. Unlike most sequence-centric or cell-centric approaches that focus on a single data type, these models embed diverse omics layers, including transcriptomic, proteomic, epigenomic, imaging, and even clinical data, into a shared latent space [112, 113].

In this context, representation and embedding are tightly coupled components of multimodal learning pipelines. Representation strategies determine how heterogeneous omics inputs are structured prior to model learning, e.g. through patch-level tokenization for imaging data, sequence-based tokenization for molecular data, or feature vectorization for tabular omics profiles. Embedding strategies, in turn, project these structured inputs into a shared latent space and align them across modalities. This alignment is typically achieved through cross-modal attention or contrastive learning, enabling both modality fusion, where complementary signals are integrated, and interaction modeling, where cross-omics dependencies for prediction or translation are learned. For example, IsoFormer integrates molecular information across omics through cross-attention to support molecular expression prediction [34], while CLOVER [33] and COMICAL [114] apply contrastive learning to enhance cross-omic consistency. Cell2Sentence [115] further extends this paradigm by integrating transcriptomic profiles, biological text, and metadata within a unified multimodal corpus, enabling more advanced biological reasoning across modalities.

Architecturally, multi-omics FMs extend beyond standard Transformer configurations as many of them incorporate hybrid architectures that integrate distinct encoding mechanisms to reason over heterogeneous biological data. HiCFoundation, for instance, uses Vision Transformer encoders to integratively model 3D genome organization [35], Life-Code combines Gated DeltaNet with a Multi-Head Self-Attention mechanism to enable knowledge transfer across modalities [41], while other models employ cross-modal attention mechanisms or contrastive components to capture mechanistic relationships among omics layers [33, 34]. More specifically, encoder-only and decoder-only designs serve different purposes in multi-omics learning. Encoder-only frameworks typically use modality-specific or unified encoders to transform transcriptomic, epigenomic, proteomic, imaging, or clinical inputs into aligned latent representations. Contrastive learning, masked modeling, and cross-attention then promote correspondence and information sharing across modalities, making these architectures suitable for multimodal integration, retrieval, classification, and sample-level representation learning. In contrast, decoder-only or encoder–decoder frameworks are more naturally suited to cross-omics translation, in which one modality conditions the generation or reconstruction of another, such as predicting gene expression from chromatin accessibility or molecular profiles from tissue images. Encoder-based integration emphasizes comprehensive representation of observed data, whereas decoder-only translation provides explicit conditional prediction but may be sensitive to modality imbalance, missing paired samples, and uncertainty in generated outputs. These biology-aware designs reflect a shift from correlational modeling toward mechanistic abstraction, where architectures are guided by biological structure and function rather than relying solely on data type and statistical associations. Overall, the multi-omics FMs have achieved promising results in regulatory inference, cancer subtyping, survival prediction, and multi-omics imputation. Their main limitations lie in the scarcity of large fully matched multimodal corpora, heterogeneity in noise and resolution across assays, and the difficulty of learning balanced representations when some modalities dominate data availability.

Viewed across the three omics paradigms, encoder-only and decoder-only architectures share a general workflow of tokenizing biological measurements, mapping them into latent embeddings, and learning contextual dependencies through self-supervised pretraining, but they differ in information flow and in the type of biological output they directly optimize [18–20]. Encoder-only models use bidirectional context and masked reconstruction to produce comprehensive representations of observed sequences, cells, or multimodal samples. They are therefore generally advantageous for annotation, classification, clustering, retrieval, integration, and other representation-centered tasks [30, 31, 33]. Decoder-only models use causal information flow and autoregressive objectives to estimate conditional distributions over biological tokens or measurements, making them more suitable for sequence design, conditional profile generation, missing-modality prediction, and cross-omics translation [20, 97]. Their native generative capability is accompanied by limitations including unidirectional context, sequential inference, error accumulation, and in cellular omics the need to impose an artificial ordering on otherwise unordered features [18, 30]. These distinctions are not absolute, as many omics models combine bidirectional encoders with autoregressive or reconstruction decoders to balance representation learning and biological generation.

Across these three paradigms, a unifying principle emerges, the abstraction of biological data into tokens, embeddings, and architectures that reflect the layered organization of living systems [18]. Sequence-centric models capture the grammatical logic of genomes, cell-centric models distill the dynamic semantics of cellular profile, and integrative frameworks synthesize complementary multimodal biological information into unified computational representations for joint analysis [116]. These approaches represent a methodological convergence toward comprehensive FMs for biology, scalable and adaptable systems capable of learning from the totality of molecular and cellular data to infer, generalize, and ultimately generate biological knowledge. The computational features of those methods are summarized in Table 2 and a more detailed conceptual guide for building omics FMs is in Section Building an omics foundation model: a conceptual guide.

**Table 2:** Summary of FMs in omics.

**Categories**	**Architecture design**	**Architecture specifics**	**Feature representation**	**Embedding Strategies**	**Model**	**Code Avail**
Sequence-centric models	Transformer-based	BERT-like (Encoder-only)	K-mer Tokenization	Token Embeddings + Positional Encoding	DNABERT [93]	Link
			K-mer Tokenization	Token Embeddings + ROPE	Nucleotide Transformer [27]	Link
			BPE Tokenization	Token Embeddings + Positional Encoding	GROVER [117]	Link
			BPE Tokenization	Token Embeddings + ROPE	GENA-LM [95]	Link
			Nucleotide-level Tokenization	Token Embeddings + Positional Encoding	SpliceBERT [118]	Link
			Nucleotide-level Tokenization	Token Embeddings + Positional Encoding	RNABERT [119]	Link
		GPT-like (Decoder-only)	K-mer Tokenization	Token Embeddings + ROPE	GENERator [120]	Link
			Non-overlapped K-mer Tokenization	Token Embeddings + Numerical Embeddings	DNAGPT [121]	Link
			BPE Tokenization	Token Embeddings + ROPE	Omnireg-gpt [97]	Link
		Multiscale Decoder-only Transformer	Nucleotide-level Tokenization	Multi-scale Token Embeddings + Positional Encoding	megaDNA [122]	Link
	Hybrid architecture	StripedHyena 2 (Hyena Convolution + Attention)	Nucleotide-level Tokenization	Token Embeddings + ROPE	Evo2 [58]	Link
		Gated DeltaNet + Multi-Head Self-Attention	Codon-level Tokenization	Token Embeddings + ROPE	Life-Code [41]	NA
		CNN + Transformer Blocks	One-hot Encoding	Convolutional Embedding + Relative Positional Encoding	Enformer [98]	Link
		CNN + Transformer (U-Net-style Hybrid)	Nucleotide-level Tokenization	Convolutional + Transformer Embeddings	AlphaGenome [99]	Link
	Other architecture	Decoder-only Hyena operator	Nucleotide-level Tokenization	Convolutional Embedding + Implicit Positional Encoding	HyenaDNA [91]	Link
		Bi-Directional Mamba Blocks	Nucleotide-level Tokenization	Reverse Complement Equivariant Token Embedding	Caduceus [92]	Link
Cell-centric models	Transformer-based	BERT-like (Encoder-only)	Rank-based Tokenization	Rank Value Embedding	Geneformer [31]	Link
			Rank-based Tokenization	Rank Value Embedding + Positional Embeddings + Contextual Encoding	Nicheformer [32]	Link
			Nonzero Peaks Sampling	Nonzero Peaks Embedding + Chromosome Embedding	EpiFoundation [100]	Link
			Rank-based Tokenization (TF-IDF-ranked cCREs)	cCRE Embeddings + Rank Embeddings + Positional Encoding	EpiAgent [101]	Link
			Bag-of-words + Gene2vec	Token Embeddings + Expression Embedding	scBERT [123]	Link
		GPT-like	Gene Name Tokenization	Token Embeddings + Expression Embedding + Condition Embedding	scGPT [30]	Link
		xTrimoGene	Un-discretized Encoding	Token Embeddings + Expression Embedding	scFoundation [102]	Link
	Hybrid architecture	scGPT + Drug-Conditional Adapters	Gene Name Tokenization	Adapter Fine-tuning	scDCA [124]	Link
	Other architecture	GeoGNNs	Spatially resolved cell–cell graph	Invariant Embeddings + Equivariant Embeddings	CI-FM [60]	Link
Integrative models	Transformer-based	GPT-like (Decoder-only)	Gene Name Tokenization	Token Embeddings + Expression Embedding + Condition Embedding	scGPT [30]	Link
		GPT-2 based Dual Encoder	SNP tokens + imaging-derived phenotypes	Contrastive Learning	COMICAL [114]	Link
		Vision Transformer	Patch-level Tokenization	Contrastive Alignment	HiCFoundation [35]	Link
		Vision Transformer + Transformer	Patch-level Tokenization for Image + Rank-based Tokenization for Gene	Contrastive Learning	OmiCLIP [112]	Link
	Hybrid architecture	Enformer + Nucleotide Transformer	K-mer for DNA and RNA + Amino-acid-level Tokenization for Proteins	Cross-modal Attention	IsoFormer [34]	Link
		Vision Transformer + Self-normalizing Network	Patch-level Tokenization for Image + Multi-omicsn Feature Vectorization	Contrastive Learning	CLOVER [33]	NA
		Gated DeltaNet + Multi-Head Self-Attention	Codon-level Tokenization	Token Embeddings + ROPE	Life-Code [41]	NA
	Other architecture	Self-normalizing network	Multi-omics Feature Vectorization	Self-normalization	SeNMo [113]	Link

**Note:** ROPE: Rotary Positional Embedding.

## Applications of omics foundation models across biological scales

FMs adapted to omics data are now applied across a wide spectrum of biological and clinical tasks, often outperforming traditional methods and enabling analyses that were previously infeasible. Trained on large-scale molecular, cellular, and spatial datasets, these models learn broad statistical and biological regularities, including sequence grammar, cellular states, cross-modal correspondences, and spatial organization, which enable them to generalize across modalities, platforms, organisms, and experimental conditions. This broad generalization capability directly mitigates the limitations of conventional task-specific models, which often struggle with data sparsity, batch variation, limited transferability, and the need for extensive labeled data. In this section, we organize the major application areas along the biological hierarchy from molecular inference to cellular modeling, tissue-level integration, and clinical translation, with detailed task definitions, representative models, and corresponding datasets summarized in Table 3. We also conclude this section with a brief synthesis of the comparative evidence currently available across these downstream applications.

**Table 3:** Application tasks and resources for omics foundation models.

**Applications**	**Specific tasks**	**Models tested**	**Training / Benchmark Datasets**
Molecular representation and network inference	Regulatory element identification	DNABERT [93], Nucleotide Transformer [27], DNAGPT [121], GROVER [117], GENA-LM [95], HyenaDNA [91], Caduceus [92], GENERator [120], OmniReg-GPT [97]	GENCODE[43], Ensembl[49], ENCODE[51], RefSeq[56]
	RNA structure & splice site prediction	RNABERT [119], SpliceBERT [118], GENA-LM [95], Life-Code [41]	RNAcentral[47], Rfam[48], GENCODE[43]
	Gene regulatory network inference	Geneformer [31], scGPT [30], scFoundation [102]	Genecorpus-30M [31], HCA [2]
	Pathway enrichment	scGPT [30], Geneformer [31]	scib [88], Single Cell Portal [59]
	SNP-phenotype association	COMICAL [114]	UK Biobank [73]
Modeling cellular states and heterogeneity	Cell type annotation & clustering	scBERT [123], scGPT [30], scFoundation [102], Geneformer [31], Nicheformer [32]	Genecorpus-30M [31], HCA [2]
	Cell-type specific expression prediction	Enformer [98], OmniReg-GPT [97], HyenaDNA [91], Caduceus [92]	ENCODE [51], FANTOM [53]
	Chromatin accessibility & dynamics	scFoundation [102], EpiFoundation [100], HiCFoundation [35]	ENCODE [51], Roadmap Epigenomics [46], HCA [2]
	Spatial niche & context inference	Nicheformer [32], CI-FM [60]	SpatialCorpus-110M [32], 10x Genomics Visium datasets [60]
Integrating tissue and multi-omics information	Cross-modality expression prediction	IsoFormer [34], Life-Code [41], OmiCLIP [112], HiCFoundation [35]	UniProt [62], PDB [65], ENCODE [51], GTEx [87]
	Multi-batch & multi-omics integration	scGPT [30], scFoundation [102], Nicheformer [32]	HCA [2]
	Cancer subtype classification via multi-omics	CLOVER [33], SeNMo [113]	TCGA [83], CPTAC [84], METABRIC [86]
Biomarker discovery and disease classification	Variant effect prediction	Nucleotide Transformer [27], Evo2 [58], Enformer [98], Caduceus [92], megaDNA [122]	ClinVar [71], dbSNP [70], gnomAD [1], GWAS Catalog [72]
	Disease diagnosis & patient stratification	Geneformer [31], CLOVER [33], SeNMo [113], COMICAL [114]	TCGA [83], UK Biobank [73], Specific clinical cohorts [132]
	Survival analysis	SeNMo [113]	TCGA [83], METABRIC [86]
Predicting perturbation and drug responses	Genetic perturbation simulation	scGPT [30], Geneformer [31], Enformer [98]	DepMap [75], Genecorpus-30M [31], ENCODE [51]
	Drug response prediction	scDCA [124], scFoundation [102]	GDSC [78], CCLE [78], LINCS L1000 [80], CTRP [79]
	Microenvironmental perturbation	CI-FM [60]	10x Genomics Visium datasets [60]
Therapeutic target identification and translational insights	Target identification & prioritization	Geneformer [31]	Genecorpus-30M [31]
	*De novo* sequence design	Evo2 [58], GENERator [120], OmniReg-GPT [97], megaDNA [122]	UniProt [62], Pfam [64], OpenGenome2 [58], RefSeq [56]

### Molecular representation and network inference

A central scientific question in omics is how to recover regulatory dependencies and functional organization from high-dimensional molecular data. At the molecular level, FMs have shown strong potential to mitigate this challenge by inferring regulatory relationships and capturing latent biological hierarchies directly from omics data. Attention mechanisms within Transformer-based FMs learn relationships between input features, such as genes or proteins, and these attention weights can be interpreted to infer regulatory networks. Geneformer, for instance, has been shown to encode gene–gene interaction hierarchies in its attention patterns during pretraining [31], suggesting that such representations implicitly reconstruct regulatory networks. In addition to attention, FM-learned embeddings can be clustered or subjected to pathway enrichment analysis, thus identifying functional modules and revealing biological pathways [125]. Moreover, models such as scGPT [30] and scFoundation [102] enable the reconstruction of gene regulatory networks and the simulation of perturbation responses, providing data-driven insights into the molecular mechanisms governing disease states and therapeutic outcomes. These approaches enable FMs to move beyond traditional statistical and network-inference methods, providing scalable and data-driven strategies for uncovering molecular interactions and pathway-level mechanisms. Although challenges in interpretability remain, the resulting representations offer valuable opportunities for linking molecular networks to cellular phenotypes.

### Modeling cellular states and heterogeneity

A core challenge in single-cell and spatial omics is to characterize cellular identity, transitional states, and context-dependent heterogeneity across tissues and disease states. Cells are dynamic and complex entities, exhibiting substantial heterogeneity across time and space, such as during distinct developmental stages or varying disease severities [126]. Investigating cellular characteristics within different microenvironments is crucial for achieving a comprehensive understanding of diseases and for uncovering potential therapeutic targets. Single-cell and spatial technologies generate high-resolution multi-omics data that reveal this heterogeneity, and FMs pretrained on large scale atlases such as the HCA [127] and the Human Tumor Atlas Network [128] are proving highly effective in annotating cell types, analyzing cellular states, and modeling dynamic trajectories. Models like scBERT, scGPT [30], Geneformer [31], and scFoundation [102] can classify cell types in scRNA-seq datasets by transferring knowledge from millions of annotated cells, substantially reducing the need for time-consuming manual annotation. The embeddings learned by these FMs encode rich biological information, allowing analysis of cellular states, developmental trajectories, and subtle transitions between populations [100].

Beyond individual cells, FMs can also model cell–cell interactions and spatial organization. CI-FM, e.g. embedded cell–cell interactions across 23 million spatially profiled cells and accurately inferred gene expression from microenvironmental context, supporting tasks such as tumor microenvironment analysis and simulating perturbation responses such as T cell infiltration [60]. Other models explicitly incorporate spatial context, with Nicheformer integrating spatial transcriptomics data to analyze cell states within their native tissue environments and to predict the spatial position of dissociated cells [32]. These developments demonstrate the capacity of FMs to model context-aware cellular identity and interaction, providing deeper insight into how cellular heterogeneity shapes tissue organization and disease processes.

### Integrating tissue and multi-omics information

A key scientific objective of multi-omics research is to connect complementary molecular layers into coherent representations of tissue organization and disease mechanisms. Moving beyond single modalities, integrative FMs aim to unify multiple omics layers, including transcriptomic, proteomic, epigenomic, and imaging data, within shared latent spaces that capture inter-modal relationships. The integrative analysis of multiple omics layers often leads to improved predictive accuracy and robustness in such tasks compared with using single-omics data alone [34, 113], as it provides complementary molecular information from different perspectives [129]. With unified multi-omics representation ability, FMs hold greater potential to advance noninvasive disease diagnosis and precision medicine. Models such as IsoFormer [34] employ cross-modal attention to fuse molecular embeddings across modalities, while OmiCLIP [112] and COMICAL [114] utilize contrastive learning to align molecular profiles with paired images. Biology-aware architectures like Life-Code [41] embed biological priors directly into the model, explicitly modeling constraints such as the central dogma to align and interpret multi-omics sequences. By aligning heterogeneous information across biological layers, these integrative models also provide better mechanistic interpretability. For instance, HiCFoundation [35] applies a Vision Transformer to model 3D genome conformation, bridging molecular structure and spatial regulation to predict epigenomic activity and further reveal regulatory loops as well as interpret links between chromatin architecture and genome function. Such integrative, biology-aware models that unify multi-omics data improve prediction and mechanistic interpretability, supporting the discovery of tissue-level disease mechanisms.

### Biomarker discovery and disease classification

From a translational perspective, a major goal of omics research is to identify molecular signatures that are predictive, biologically interpretable, and clinically actionable. At the level of tissues and organisms, FMs are increasingly applied to biomarker discovery and disease classification. FMs facilitate biomarker discovery by learning molecular features that are predictive of disease states from high-dimensional omics data. For example, scPRINT [130] demonstrates the potential of FMs for biomarker-related discovery by inferring cell-specific gene networks from more than 50 million single-cell profiles and identifying disease-related pathways and candidate molecular signatures in specific cellular contexts. In clinically oriented multi-omics settings, models such as SeNMo [113] learn cross-omics representations from TCGA datasets and uncover prognostic molecular signatures associated with cancer survival outcomes. Similarly, contrastive and graph-based models such as CLOVER [33] and CI-FM [60] identify biologically meaningful features across modalities and spatial contexts, capturing tumor heterogeneity and microenvironmental structure. These approaches reduce reliance on manual feature engineering by learning transferable and biologically meaningful feature representations. Leveraging these learned representations, FMs further support disease classification by modeling relationships among molecular, cellular, and spatial features. For instance, SeNMo achieves accurate cancer type prediction and survival stratification, while CLOVER and CI-FM enable robust tumor classification and spatial characterization across datasets. By capturing nonlinear relationships among genes, proteins, and clinical variables, FMs improve both diagnostic performance and generalization, highlighting their potential for data-driven precision medicine.

### Predicting perturbation and drug responses

Another major scientific question in omics is whether learned representations can predict how cells and tissues respond to genetic or pharmacological perturbations. FMs have been increasingly applied to predict cellular and molecular responses to such interventions. scGPT [30] and Geneformer [31] can simulate gene knockouts or drug treatments, predicting transcriptomic changes *in silico*. scDCA [124] extends this capability through adapter-based fine-tuning, achieving strong generalization even to unseen cell lines. Similarly, scFoundation [102] predicts cellular responses to chemical perturbations and drug treatments, leveraging its large-scale pretrained representations to identify potential therapeutic outcomes. These models enable virtual screening and hypothesis generation, helping prioritize experiments and reduce laboratory workload. Although computational predictions still require experimental validation, the scalability of FMs provides a powerful framework for modeling perturbation dynamics across diverse biological contexts.

### Therapeutic target identification and translational insights

Beyond prediction, an important translational question is whether omics FMs can help move from disease-associated signals to actionable therapeutic hypotheses. FMs support therapeutic target identification and translational research by linking disease-associated signatures to potentially actionable biological mechanisms. By integrating large-scale multi-omics data with disease phenotypes, these models can uncover regulators and pathways that are not only associated with pathogenesis but also potentially suitable for therapeutic intervention. For instance, Geneformer [31] has been used to predict novel cardiomyopathy targets and further supported these predictions with experimental validation in engineered cardiac microtissues. In parallel, models such as scGPT [30], scFoundation [102], and CellFM [131] further extend this translational potential by enabling perturbation-response prediction, reverse perturbation analysis, and drug-response modeling, thereby supporting the prioritization of candidate therapeutic interventions. In addition to target discovery, FMs can assess target druggability by integrating structural, functional, and pharmacogenomic information. This enables prioritization of therapeutically actionable genes and pathways, providing a computational foundation for precision drug development. Although direct *in vitro* or *in vivo* validation remains limited in the current omics FM literature, these studies collectively suggest that FMs can increasingly support mechanism-guided therapeutic discovery and facilitate the translation of omics findings into precision medicine applications.

Taken together, these application studies also provide an emerging, although still fragmented, basis for comparing the strengths and limitations of current omics FMs across downstream settings.

At present, however, the omics FM literature still lacks a fully standardized benchmarking framework across model classes, datasets, preprocessing pipelines, and evaluation metrics. As a result, direct cross-model comparison remains difficult, and the evidence discussed here is best interpreted as study-level comparative evidence synthesized from the original literature rather than as a unified head-to-head benchmark.

Even so, several original studies already provide informative comparative signals. Geneformer [31] reported improved cell-type annotation and robust cross-platform transfer, while Nicheformer [32] outperformed multiple existing baselines in spatial-context modeling. In multimodal tissue-level analysis, OmiCLIP/Loki [112] showed strong performance across tasks including tissue alignment, annotation, retrieval, and expression prediction, and scPRINT [130] achieved competitive or superior results in gene-network inference. Collectively, these studies suggest that current omics FMs are particularly promising for transfer learning, spatial-context modeling, and cross-modal representation learning, while also underscoring the need for more standardized benchmarks for fair comparison across models and tasks.

## Building an omics foundation model: a conceptual guide

**Figure 3:**
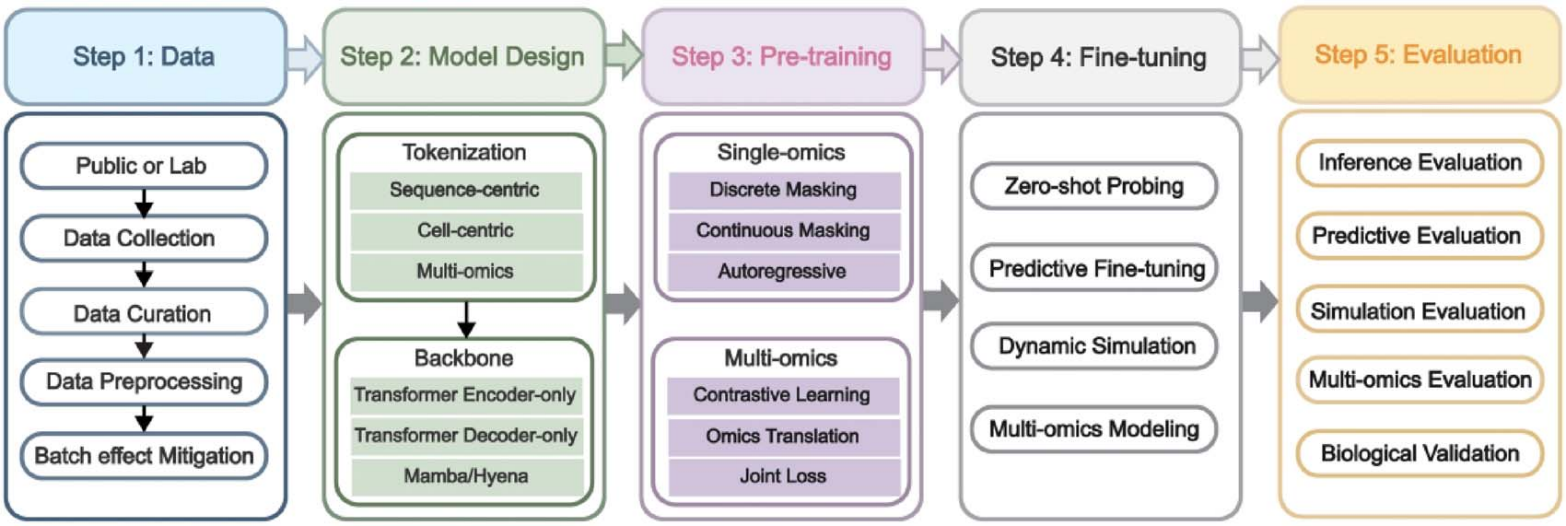
Conceptual pipeline of building an omics FM, summarizing the major steps from data acquisition to evaluation.

Using a Transformer-based model as an example, this section provides a conceptual overview of omics FM training, outlining the step-by-step process and highlighting key considerations at each step. While alternative architectures such as Mamba and Hyena exist, their differences from Transformers are primarily confined to the model design stage (Step 2); the remaining steps follow the same conceptual pipeline regardless of the backbone architecture. The overall conceptual pipeline is shown in Fig. 3.

### Step 1: Data acquisition, curation, and preprocessing

The process begins with assembling large-scale datasets, ranging from single-omics cohorts to multi-omics collections. In the multi-omics setting, both paired and unpaired datasets should be collected—paired samples, where multiple omics are measured on the same biological specimen, provide direct cross-modal supervision, whereas unpaired cohorts substantially increase the available scale. In practice, datasets are obtained from major public repositories such as TCGA [83], HCA [2], HPA [3], UK Biobank [73], ENCODE [51], GEO [57], and CPTAC [84], as well as in-house laboratory experiments or private clinical datasets. After acquisition, the collected datasets require systematic curation. This includes quality control procedures tailored to each omics type, ensuring consistent feature identifiers (e.g. gene symbols, protein IDs) across datasets, and consolidating essential metadata such as sample annotations, experimental conditions, and clinical outcomes. At this stage, samples or features with excessive missingness or clearly inadequate data quality are typically removed.

Finally, preprocessing transforms the curated data into model-ready inputs, with procedures that differ substantially across omics modalities. For sequence-centric omics, preprocessing is relatively streamlined. Genomic models are typically trained on reference genome segments or curated multi-species genome corpora partitioned into fixed-length windows, while assay-derived regulatory data such as ChIP-seq, ATAC-seq, or CAGE require converting raw measurements into genomic targets such as peaks, coverage tracks, or base-resolution profiles, which are then paired with the corresponding genomic sequences for model training. At the protein level, a common preprocessing step is redundancy reduction through sequence-identity clustering or homology-aware splitting, which helps limit train–test leakage and overestimation of generalization. In cell-centric omics, preprocessing involves more extensive modality-specific pipelines. In single-cell transcriptomics, the process usually begins with cell filtering based on quality metrics including mitochondrial gene fraction, the number of detected genes, and total UMI counts. A key design choice at this stage is normalization: while traditional workflows apply library-size normalization followed by log-transformation, many FMs intentionally bypass this step and operate directly on raw counts to avoid the compositional biases introduced by standard normalization, for instance using rank-based encoding as in Geneformer or value binning as in scGPT. Additionally, most models select a subset of highly variable genes to reduce input dimensionality, although some architectures retain the full transcriptome at the cost of increased computation. The spatial transcriptomics workflow largely mirrors that of scRNA-seq; the main additional requirement is normalizing spatial coordinates across slides, as the physical extent and coordinate range can vary substantially between tissue sections. For multi-omics data, preprocessing generally follows the modality-specific procedures described above, applied independently to each omics layer before integration.

### Step 2: Model architecture design

After data curation, the next critical design choice for Transformer-based model is selecting an input tokenization strategy that matches the omics data structure and modeling goals. In discrete, sequence-centric omics such as DNA, RNA, or protein, NLP-style tokenization with k-mers or residues is a standard and effective choice [133]. In contrast, cell-centric omics are typically represented as continuous, unordered profiles, and there are two main ways to turn them into model inputs. One option is to rank biomarkers by their expression levels to form a discrete sequence [31, 32], at the cost of discarding part of the quantitative signal. An alternative is to retain expression values explicitly, either by mapping them through an MLP or by discretizing them with a value-binning scheme while embedding biomarker identities separately, as in scGPT [30]. Notably, the extreme sparsity of scRNA-seq data poses distinct challenges for each strategy. Value-based tokenization with mean squared error loss is susceptible to collapse toward trivial zero predictions, whereas rank-based tokenization with cross-entropy loss is more robust to sparsity but discards quantitative magnitude information. In multi-omics settings, a common strategy is to tokenize each modality separately using omics-specific schemes; in cases where cross-omics relationships need to be modeled, the tokenizer itself can be pretrained on multi-omics data so that it captures cross-omics dependencies, as in [41].

Building on the chosen tokenization, the model backbone for single-omics should reflect the structure of the resulting profiles. For discrete, sequence-centric omics data, Transformer architectures are a natural choice: encoder-only models with masked language modeling are suited for representation learning, while decoder-only models with next-token prediction (NTP) support generative tasks. Beyond Transformers, sub-quadratic architectures such as Hyena [134] and Mamba [135] have emerged as attractive alternatives for extremely long sequences, replacing self-attention with long convolutions or selective state-space mechanisms that scale sub-quadratically or linearly with sequence length. These architectures can process raw continuous signals directly and may not require explicit tokenization. In contrast, continuous cell-centric omics are typically represented as unordered profiles and therefore align poorly with decoder-only architectures trained with NTP. As a result, most current models adopt encoder-only designs with masked modeling or encoder–decoder architectures that reconstruct the full profiles. However, how to exploit autoregressive training in a principled way, rather than relying on NTP for such unordered cell-level data, remains an open question, with approaches like scGPT [30] offering only initial explorations.

In multi-omics scenarios, architectural choices are tightly coupled to the pretraining objective. When the primary aim is representation alignment or fusion, a typical choice is to use multiple omics-specific encoders in parallel and combine their outputs through cross-attention blocks or a fusion encoder, often reusing pretrained single-omics backbones. In cases where the emphasis shifts to modeling omics interactions or cross-omics generation, architectures either retain multiple omics-specific encoders with cross-modal decoders or collapse to a single primary encoder that takes one omics as input and predicts another. A third option is to use a unified transformer encoder that jointly ingests tokens from multiple omics within a single backbone, typically with omics-specific decoders [136].

### Step 3: Self-supervised pretraining

Once the architecture is defined, the core engine of FMs is self-supervised pretraining, which allows the model to learn biological signals from massive unlabeled datasets. Similar to model design, the choice of pretraining objective is tightly coupled with the data structures and pretraining purpose.

Self-supervised pretraining in single-omics models broadly falls into two major objective families: masked modeling and autoregressive modeling. Masked modeling—typically paired with encoder-only architectures—applies to both discrete omics sequences and continuous profiles. When the input admits an ordered tokenization, either inherently (DNA, RNA, protein) or through expression-based ranking (scRNA-seq), a subset of tokens is masked and recovered with a cross-entropy loss. For truly continuous profiles without discretization, the same principle reduces to masking biomarker values and reconstructing them using a regression loss, typically mean squared error. Autoregressive pretraining constitutes the second family, commonly realized in decoder-only architectures for generative modeling and capturing conditional dependencies. For ordered sequences (native or rank-based discretizations), autoregressive training is straightforward: each token is predicted from its predecessors. However, continuous omics profiles lack an intrinsic ordering, rendering autoregressive factorizations ill-defined. One workaround, explored by scGPT [30], is to iteratively generate unknown expression values conditioned on known ones until the full profile is recovered.

Transitioning to multi-omics settings, pretraining objectives can be grouped according to the type of cross-modal relationship they aim to capture. For objectives centered on representation alignment, contrastive learning—typically implemented with separate encoders for each omics modality—remains the primary strategy. These separate encoders preserve each modality’s native feature space, while contrastive losses pull together embeddings of paired samples to impose cross-omics consistency. Beyond alignment, reconstruction-based or masked modeling tasks can be incorporated to strengthen modality-specific representations; when combined with contrastive learning, these objectives are often jointly optimized through a multi-task loss to balance alignment with within-modality signal recovery. When the goal shifts to modeling explicit interactions across omics, pretraining is commonly formulated as cross-omics translation, where the model predicts one omics from another using regression- or likelihood-based losses. Finally, unified transformer formulations take a different approach: instead of modality-specific encoders, they rely on masked modeling losses applied to mixed-omics token sequences, with tokens from multiple modalities partially masked and jointly recovered within the same backbone.

In practice, the computational resources required for pretraining are largely determined by the per-sample token length. Models that process long sequences, such as DNA or single-cell FMs encoding the entire gene vocabulary, face quadratic memory complexity in standard self-attention and typically require multi-GPU or multi-node training. In contrast, modalities with shorter token sequences, such as targeted spatial transcriptomics panels, can often be trained on less GPUs. For multi-omics models, hardware demands depend on the specific modality combination, primarily driven by the longest-sequence modality involved. Notably, sub-quadratic architectures such as Mamba, which scales linearly with sequence length, can substantially alleviate these hardware requirements for long-sequence modeling.

### Step 4: Fine-tuning for downstream tasks

After pretraining, FMs are usually adapted to downstream applications generally through four broad classes of task paradigms, each partially corresponding to the applications outlined in Section Applications of omics foundation models across biological scales. The first two paradigms focus on static inference and prediction. Zero-shot probing leverages the intrinsic structure of the pretrained representation without updating model parameters, which is primarily applied to tasks centered on Molecular Representation and Network Inference. For example, the regulatory networks can be inferred by analyzing the attention weights of pretrained FMs, and the pathway-level functional modules can be identified through embedding clustering and similarity analysis [30, 31]. In parallel, predictive fine-tuning trains a supervised head to map representations to phenotypic labels, supporting tasks that require precise prediction including Modeling Cellular States, Biomarker Discovery, and Disease Classification. Specifically, classification heads can be attached for discrete outcomes such as cell type annotation [30], disease diagnosis, or tissue niche categorization [32], while regression heads can be employed for continuous targets, such as microenvironment-informed expression prediction [18] or cancer survival forecasting. In the context of biomarker discovery, the same predictive heads can be fine-tuned and biomarkers are subsequently identified by ranking features according to their learned importance scores.

Moving beyond static labeling, dynamic simulation modeling extends by predicting how cellular states evolve under genetic or chemical perturbations. This paradigm aligns with applications such as Predicting Perturbation and Drug Responses, where models aim to simulate transcriptomic changes following gene knockouts or drug treatments. Rather than producing scalar predictions, these tasks rely on conditional decoding strategies to generate high-dimensional counterfactual expression profiles that reflect the expected trajectory of the perturbed state.

The final paradigm, multi-omics modeling, addresses tasks that require integrating heterogeneous biological layers. It supports applications such as Integrating Tissue and Multi-Omics Information and Therapeutic Target Identification, where the objective is to align omics information or capture cross-omics interactions. Fine-tuning strategies commonly include contrastive approaches that align representations across modalities and translation heads that generate missing omics layers. In translational settings, the aligned representations can be further leveraged using ranking-based fine-tuning to prioritize therapeutically actionable targets based on aggregated molecular and clinical evidence.

Together, the above task paradigms, except for zero-shot probing, essentially follow a supervised fine-tuning framework, where explicit downstream objectives and corresponding label signals are required. The specific form of supervision varies across paradigms: for instance, cross-entropy loss is commonly used for discrete classification tasks such as cell-type annotation and disease diagnosis, while mean squared error is employed for continuous regression targets such as perturbation response prediction and survival forecasting. Under this framework, full fine-tuning updates all parameters of both the pretrained backbone and the task-specific head (e.g. classification or regression layers), and generally achieves the best performance. However, the computational cost of updating entire FMs can be prohibitive, particularly for experimentally oriented laboratories with limited GPU access. In principle, full fine-tuning requires comparable GPU memory to pretraining for the same model, as all parameters and their gradients must be stored, with the actual requirements further modulated by the batch size. To alleviating this challenge, parameter-efficient fine-tuning (PEFT) strategies offer a practical alternative by introducing a small number of additional trainable parameters to adapt the model to downstream tasks, while keeping the majority of pretrained parameters unchanged. Representative PEFT methods include Low-Rank Adaptation (LoRA) [137] and Adapter tuning [138]. LoRA injects pairs of low-rank decomposition matrices alongside the original weight matrices of the backbone, and only these low-rank matrices are updated during fine-tuning; at inference time, they can be merged back into the original weights with no additional latency. Adapter tuning, by contrast, inserts small trainable bottleneck modules between the frozen transformer layers, which learn task-specific transformations while leaving the backbone entirely unchanged. These strategies substantially reduce GPU memory requirements, making it feasible to fine-tune large FMs on a single consumer-grade GPU. Other PEFT strategies, such as prompt tuning, also exist but have seen limited adoption in this domain to date

### Step 5: Evaluation and validation

Building on the fine-tuning strategies above, we organize evaluation into the same task-aligned categories. In static inference settings, assessment depends on the task type. Zero-shot network inference, where ground-truth labels are often absent, typically relies on biological concordance, such as quantifying the overlap between inferred interactions and experimentally validated databases. For predictive fine-tuning, performance is summarized using standard supervised metrics: classification tasks (e.g. cell-type annotation, disease classification) are assessed by accuracy, F1, and AUROC, whereas regression tasks (e.g. expression prediction) are evaluated using $R^{2}$, Pearson/Spearman correlation, and MAE/RMSE. Additionally, survival analysis is usually evaluated by the Concordance Index (C-index) and time-dependent AUROC to validate risk stratification.

In dynamic simulation settings, tasks typically require evaluating high-dimensional reconstruction fidelity. Standard reconstruction errors such as MSE and correlation coefficients provide a basic assessment, but distributional metrics including the Wasserstein distance and Maximum Mean Discrepancy are equally important for determining whether the simulated cellular states, e.g. those produced under drug perturbation, align statistically with real biological distributions rather than merely reducing pointwise error.

A separate category concerns multi-omics modeling, where the evaluation focus shifts toward ranking quality and cross-omics fidelity. Within this multi-omics setting, Therapeutic target identification is treated as a prioritization problem and evaluated using ranking-based metrics such as AUROC, Top-(k) Hit Rate, and Normalized Discounted Cumulative Gain, which quantify the model’s ability to retrieve actionable targets. Evaluation of Integrating Tissue and Multi-Omics Information is further separated into cross-omics alignment and omics translation. Alignment quality is measured by cross-modal retrieval accuracy between paired modalities, with optional analyses examining modality contributions—e.g. by ranking or weighting their relative importance. In contrast, translation performance is assessed using reconstruction metrics such as MSE or Pearson correlation to determine whether the predicted profiles faithfully capture the corresponding biological signals.

Beyond these task-level metrics, evaluation of omics FMs should also include omics-specific and FM-aware validation criteria. In single-cell and multi-batch settings, this is increasingly supported by scIB-style metrics that jointly assess batch removal and biological conservation, including measures such as kBET, graph iLISI, batch ASW, graph connectivity, ARI, NMI, and cell-type ASW. In addition, because FMs are expected to learn transferable representations rather than only task-specific predictors, evaluation should extend beyond fine-tuned downstream accuracy to zero-shot performance, linear probing, and transfer across cohorts, platforms, tissues, or perturbation settings. Recent studies have shown that such settings can reveal limitations that are not apparent from conventional fine-tuning alone. When applicable, model outputs should also be examined for biological plausibility, e.g. by testing whether embeddings or predictions preserve canonical cell-type markers, pathway activity patterns, or experimentally supported regulatory relationships [32, 88, 139].

Crucially, fair assessment of omics FMs requires carefully designed benchmarking protocols rather than isolated case studies. Models should be compared against strong non-foundation baselines as well as peer FMs on standardized datasets that cover the main application areas summarized in Section Applications of omics foundation models across biological scales, using shared splits and clearly reported data preprocessing steps. To properly reflect the fine-tuning strategies discussed above, evaluations should report both full fine-tuning and PEFT strategies, and include ablations that disentangle the contributions of the backbone, task-specific heads, and PEFT modules. When applicable, studies should also examine whether model outputs remain biologically plausible—e.g. whether they preserve canonical cell-type markers, gene and pathway signatures, or clinically established biomarkers. Comparisons should also incorporate robustness checks (e.g. cross-cohort transfer, platform shifts, out-of-distribution tissues or perturbations) and sensitivity analyses with respect to training data size or label noise.

## Challenges and future directions

The application of FMs to omics research represents a paradigm shift, yet the field is currently navigating a critical transition from proof-of-concept to robust utility. While FMs offer unprecedented capabilities in representation learning, their widespread adoption is hindered by substantial hurdles related to computational costs, interpretability, data privacy, and the capability to model complex biological dynamics. Alleviating these challenges is not merely about refinement but requires fundamental shifts in architectural design and evaluation ecosystems. Here, we synthesize the primary obstacles and the emerging research directions poised to overcome them.

### Efficiency and scalability

A primary barrier to the democratization of omics FMs is the prohibitive computational expense. Training large-scale models from scratch on terabyte-scale genomic or single-cell datasets demands high-end GPU or TPU clusters and extended processing times, rendering such endeavors inaccessible for many research groups [22]. While fine-tuning offers a partial remedy, even this process remains resource-intensive for multi-billion-parameter models.

To address this, the field must pivot toward fundamentally more efficient modeling paradigms. Two key directions are emerging. First, there is a need to move beyond standard Transformer backbones, which suffer from quadratic memory complexity with respect to sequence length. Future research should actively explore linear-complexity architectures, such as state space models (e.g. Mamba) [92, 135] and implicit convolution models like Hyena [91, 94]. These architectures promise to model ultra-long genomic sequences and high-resolution spatial contexts without the quadratic memory overhead of attention mechanisms.

Second, to mitigate the cost of adaptation, the community is increasingly adopting PEFT strategies. Techniques such as LoRA and adapters allow for targeted local updates on shared backbones without retraining full models [137, 140, 141]. Recent adaptations like scDCA demonstrate that lightweight components can achieve competitive performance even in data-limited scenarios [124]. We envision a future dominated by modular FMs—characterized by a robust pretrained core coupled with plug-and-play, modality-specific adapters—significantly enhancing reusability and reducing the economic barrier to entry.

### Privacy-preserving and federated omics

As FMs scale to include clinical multi-omics cohorts (e.g. patient data from hospitals), data privacy becomes a prohibitive bottleneck alongside computational costs. Regulations such as GDPR and HIPAA restrict the centralization of sensitive genomic and clinical data, preventing the creation of the massive, diverse datasets required to train truly robust clinical FMs. Current models are largely trained on de-identified public research data, which often lacks the clinical granularity and diversity of real-world patient populations.

To unlock the value of siloed clinical data, the development of *Federated Foundation Models* is imperative. The success of Tabula [142] demonstrates the feasibility of privacy-preserving FM pretraining in decentralized single-cell settings, although large biomedical FMs trained in this manner remain rare. Future architectures must therefore support federated learning (FL), in which models are trained collaboratively across decentralized institutions (e.g. hospitals) without transferring raw data off-site. This setting also requires additional safeguards, such as differential privacy, to reduce the risk of model inversion and related privacy attacks.

Beyond conventional FL, split learning provides another privacy-aware distributed training paradigm for biomedical FMs [143]. In split learning, a neural network is partitioned at a cut layer, such that early layers are executed locally and only intermediate activations are transmitted to a central server for subsequent computation and backpropagation. Compared with FL, which typically maintains a full local model at each client and exchanges model updates, split learning can reduce local computational burden and limit direct sharing of raw omics data, making it particularly attractive for clinical multi-omics settings with constrained institutional hardware [144]. However, because intermediate representations may still leak sensitive information, practical split-learning frameworks may require additional protections, such as differential privacy, secure aggregation, or cryptographic safeguards [145].

### Challenges for clinical translation

While privacy-preserving and FL strategies are essential for enabling cross-institutional training, privacy is only one of several barriers to the clinical translation of omics FMs. In real-world clinical settings, these models must also contend with limited cohort sizes and demographic imbalance in publicly available omics resources. Alleviating these constraints requires targeted technical solutions for data-efficient adaptation, privacy-preserving collaboration, and equitable generalization across populations.

Major obstacle to clinical translation is that patient-derived omics data are highly privacy-sensitive and are usually siloed across institutions, which limits the feasibility of centralized large-scale model training. Accordingly, privacy-preserving collaborative learning has become an essential technical direction for clinical omics FMs. Federated and split learning provide practical privacy-preserving solutions to this challenge [145–147].

A further barrier to clinical translation is the limited sample size of many clinical omics cohorts, especially in rare diseases, narrowly defined patient subgroups, or single-center studies. In such settings, full end-to-end training or exhaustive fine-tuning of large models is often impractical and may increase the risk of overfitting [148, 149]. A feasible direction is to combine large-scale self-supervised pretraining with transfer learning and parameter-efficient adaptation, so that pretrained models can be specialized to downstream clinical tasks using only limited local data. Recent studies on pretrained single-cell FMs such as scGPT [30] and scPEFT [150] further suggest that adapter-based and other PEFT strategies can improve adaptation to new biological contexts while substantially reducing the number of updated parameters, highlighting their potential for low-resource clinical deployment.

Another major challenge is the demographic imbalance of publicly available omics resources, which remain heavily skewed toward populations of European ancestry. Such imbalance can limit model generalizability in underrepresented populations and may bias downstream biomarker discovery, reference annotation, and clinical interpretation. Recent studies have shown that transcripts from non-European samples remain underrepresented in current reference gene annotations, while equitable machine learning frameworks such as PhyloFrame demonstrate that population-aware modeling can improve predictive performance across ancestries [151, 152]. Accordingly, future omics FMs should place greater emphasis on ancestrally diverse cohort construction, population-aware reference resources, and subgroup-stratified evaluation to reduce the risk of amplifying existing inequities in precision medicine [153].

### Interpretability and trust

The ”black box” nature of deep learning poses a unique risk in biomedicine. FMs are often criticized for their lack of transparency, making it difficult to distinguish between genuine biological signal and artifacts [18]. This issue is compounded by the risk of ”hallucination,” where models generate statistically plausible but biologically factually incorrect outputs (e.g. inventing nonexistent regulatory interactions) [42]. For clinical applications, such opacity is unacceptable; understanding the reasoning behind a drug response prediction or subtype classification is essential for establishing trust and ensuring patient safety.

While *post hoc* analysis methods like attention visualization, SHAP, and LIME provide some insight [154–157], the future lies in intrinsic interpretability. Research must prioritize ”biology-aware” architectures that embed scientific priors directly into the model structure [158]. By integrating pathway databases, gene regulatory networks, and protein interaction maps into the attention mechanisms or graph structures—similar to approaches used in DeePathNet [104]—models can be constrained to generate predictions that align with established biological knowledge. This synergy between data-driven learning and mechanistic priors will be crucial for transforming FMs from predictive engines into engines of discovery that generate verifiable biological hypotheses.

### Beyond correlation: causal and mechanistic reasoning

Closely linked to interpretability is the challenge of causality. A fundamental limitation of current omics models is that, despite architectural advances from BERT-style DNA language models (DNABERT [93], Caduceus [92]) and long-range regulatory predictors (Enformer [98]) to cell-centric transformers (Geneformer [31], scGPT [30]), they remain fundamentally correlation-based learners that capture statistical associations rather than causal mechanisms. While an FM might correctly predict that Gene A and Gene B are co-active, it often fails to distinguish whether A regulates B, B regulates A, or both are regulated by a confounder C. This ”causal confusion” limits the utility of FMs in identifying therapeutic targets, where intervening on a downstream effect rather than an upstream driver yields no clinical benefit.

The next generation of omics FMs must integrate *Causal Representation Learning*. Broadly, causal representation learning seeks to recover latent variables with causal semantics from high-dimensional observations, so that models can capture stable mechanisms rather than spurious correlations [159]. In contrast to purely observational learning, this paradigm leverages signals such as interventions, mechanism shifts, and distribution changes to identify causally meaningful factors and improve robustness across environments [160, 161]. This perspective is particularly relevant to omics, where single-cell perturbation data, CRISPR/Perturb-seq experiments, and drug-response screens naturally provide interventional settings for learning mechanistically grounded representations. More broadly, recent perspectives have argued that perturbation atlases may serve as a foundation for causal cell and tissue biology, further underscoring the relevance of causal representation learning for future omics FMs [162, 163].

Future research should focus on hybrid architectures that incorporate structural causal priors, intervention-aware objectives, or mechanism-invariant constraints into FMs for omics. Rather than only reconstructing masked inputs (e.g. hidden genes), benchmarking should increasingly emphasize perturbation response prediction, causal effect estimation, and the ability to generalize across biological contexts. Methods that explicitly model the logic of interventions—rather than only observational distributions—will be essential for transforming FMs from descriptive tools into engines of mechanistic discovery.

### Temporal dynamics and 4D omics modeling

Biological systems are inherently dynamic, governed by continuous processes such as development, aging, and disease progression. However, the vast majority of training data for cell-centric models like Geneformer [31] and scGPT [30] consists of static snapshots (e.g. single-cell RNA-seq atlases) that lack temporal labels. Consequently, current models learn stationary manifolds of cellular states but struggle to infer the vector fields—velocity, acceleration, and trajectory—that drive cells from one state to another. Applying static FMs to dynamic problems often results in disjointed predictions that fail to capture the continuous flow of biological time.

Future work must bridge the gap between static data and dynamic modeling through *Temporal Foundation Models*. A promising avenue lies in the integration of Neural Ordinary Differential Equations (Neural ODEs) with Transformer backbones, allowing models to learn continuous-time latent dynamics from discrete snapshot data. Recent innovative approaches include Chronocell [164], which reconstructs cellular event sequences using advanced mathematical modeling techniques, and GeneTrajectory [165], which identifies gene trajectories by calculating the optimal transport distance between gene distributions on the cell–cell graph. By explicitly modeling the derivative of the cellular state with respect to time, these ”4D FMs” could forecast future disease states or developmental trajectories for individual patients, moving beyond static classification to predictive prognosis.

### Standardization and robust evaluation ecosystems

Finally, the rapid proliferation of omics FMs has outpaced the development of rigorous evaluation protocols. The current literature is fragmented, lacking standardized benchmarks, consensus datasets, and uniform metrics, which makes objective comparison between models exceedingly difficult [18]. Furthermore, models often struggle with generalization and robustness when applied to data from different populations, sequencing platforms, or experimental batches [166].

To mature as a field, the community must establish a standardized evaluation ecosystem. Initiatives such as the Open Problems in Single Cell Analysis benchmark represent vital first steps [82]. Future efforts must expand these frameworks to multi-omics and spatial domains, defining consensus tasks that rigorously stress-test models. Evaluations should go beyond simple accuracy metrics to assess robustness against technical variability (e.g. batch effects) and biological plausibility. A transparent, reproducible benchmarking culture is essential to identify true methodological progress and guide the responsible deployment of these powerful tools.

## Conclusion

This survey has systematized the rapidly evolving landscape of FMs in omics research. By distinguishing between sequence-centric, cell-centric, and multi-omics architectures, we have clarified how these models encode the hierarchical logic of biology—from the grammar of genomic sequences to the complex phenotypes of tissues. The evidence suggests that FMs have successfully moved beyond traditional feature engineering, offering a scalable approach to navigate the high dimensionality and sparsity inherent in molecular data.

However, translating these computational capabilities into reliable biomedical utility requires navigating a difficult transition. As we have detailed, the current generation of models faces structural limitations regarding computational efficiency, interpretability, and the risk of hallucination. The path forward is not simply to train larger models, but to design biologically grounded ones. The future of this field lies in three critical shifts: moving from correlation to causality to support mechanistic reasoning; transitioning from static snapshots to dynamic, temporal modeling; and integrating models directly into the experimental loop to validate predictions autonomously.

Ultimately, the success of omics FMs will not be measured solely by their performance on computational benchmarks, but by their ability to generate hypotheses that withstand experimental verification. As the community addresses the challenges of standardization and privacy, these systems are poised to transform omics from a descriptive science into a predictive and actionable discipline. This evolution represents a vital step toward bridging the gap between data-driven inference and the rigorous demands of precision medicine.

**Key Points**

- Foundation models represent a fundamental departure from conventional machine learning, leveraging self-supervised pretraining to extract transferable representations that effectively handle the high dimensionality and sparsity of biological data.
- A comprehensive review of 29 emerging FM methodologies is presented, systematically categorizing architectures into three distinct paradigms: sequence-centric (e.g. DNA/protein), cell-centric (e.g. scRNA-seq), and multi-omics integration.
- To facilitate transparent benchmarking and accelerate progress, we compile a comprehensive catalog of publicly available omics datasets and standardized evaluation frameworks, summarizing 47 datasets and resources essential for next-generation model development.
- A detailed technical analysis of the model development pipeline is provided, examining variations in tokenization schemes, pretraining objectives, and fine-tuning strategies across applications ranging from biomarker discovery to perturbation response prediction.
- Critical challenges regarding computational efficiency, interpretability, and causality are synthesized to propose actionable future directions, including the development of biology-aware architectures, temporal dynamics modeling, and lab-in-the-loop autonomous experimentation.

## Data Availability

All available omics datasets and resources are summarized in Table 1.
